# Targeting angiogenesis: advances in the design and engineered applications of nanobodies

**DOI:** 10.3389/fimmu.2026.1912560

**Published:** 2026-08-21

**Authors:** Yuxuan Liu, Zhaohai Li, Yuanyuan Ma, Yulin Liu

**Affiliations:** 1 No.2 Research Laboratory, Changchun Institute of Biological Products, Changchun, Jilin, China; 2 School of Engineering, China Pharmaceutical University, Nanjing, Jiangsu, China

**Keywords:** angiogenesis, engineering strategies, nanobodies, theranostic, translational applications

## Abstract

Angiogenesis is a fundamental physiological process; however, its pathological dysregulation drives diseases such as solid tumors, wet age-related macular degeneration, and rheumatoid arthritis. Although clinically effective, conventional anti-angiogenic monoclonal antibodies are limited by poor tissue penetration, off-target toxicities, and susceptibility to compensatory resistance. This review systematically examines the structural advantages and engineering strategies of nanobodies (Nbs) targeting angiogenesis-related pathways. Advanced engineering approaches, such as AI-assisted humanization and multivalent assembly, effectively mitigate immunogenicity and extend serum half-life. Furthermore, multispecific designs can simultaneously block compensatory pathways to circumvent resistance. Moreover, preclinical studies indicate that integrating these molecules into site-specific nanobody-drug conjugates and targeted delivery vehicles may improve therapeutic precision and local drug accumulation; however, their long-term safety, manufacturability, and clinical benefit remain to be established. Functionalizing Nbs with radionuclides or fluorophores may enable the development of novel theranostic platforms that support real-time molecular imaging and image-guided surgery. In parallel, nanobody-based CAR-T (Nb-CAR-T) cells facilitate the targeted remodeling of the disease microenvironment. Ultimately, this review highlights the value of engineered Nbs as a highly programmable and transformative platform. By overcoming key limitations of conventional antibodies, engineered nanobodies open new avenues for precise, multi-dimensional interventions in solid tumors. Their potential in certain non-neoplastic angiogenic diseases is emerging but requires further validation.

## 1 Introduction

Angiogenesis, the formation of new blood vessels from the pre-existing vasculature, is a critical mechanism for maintaining organismal homeostasis and tissue repair. Although indispensable for biological events such as embryonic development, tissue growth, and wound healing, this process requires strict spatiotemporal regulation. Its persistent, aberrant activation serves as a common driver for numerous angiogenesis-dependent diseases, including solid tumors, wet age-related macular degeneration (wAMD), diabetic macular edema (DME), and rheumatoid arthritis (1, 2) (**Table 1**).

**Table 1:** Common angiogenesis-dependent diseases and pathological mechanism.

Disease category	Related diseases	Pathological features and angiogenesis mechanisms
Malignant Tumors	Colorectal cancer, Non-small cell lung cancer, Hepatocellular carcinoma, Glioblastoma	Hypoxia in the tumor microenvironment induces overexpression of vascular endothelial growth factor(VEGF), driving abnormal angiogenesis to supply nutrients and promote metastasis (3–5).
Ocular Diseases	Wet age-related macular degeneration (wAMD), Diabetic macular edema (DME)	Abnormal leakage and hemorrhage of choroidal/retinal neovascularization lead to photoreceptor damage and vision loss (6, 7).
Chronic Inflammation	Rheumatoid arthritis (RA), Psoriasis	Vasculature formation in synovial tissue, inflammatory cell infiltration, and increased vascular permeability cause tissue swelling and destruction (8, 9).
Fibrotic Diseases	Idiopathic pulmonary fibrosis, Liver fibrosis	Abnormal communication between blood vessels and stromal cells promotes fibroblast activation and extracellular matrix (ECM) deposition (10, 11).
Vascular Malformations	Infantile hemangioma, Arteriovenous malformation	Abnormal proliferation of local vascular endothelial cells, high expression of Glucose Transporter 1 and other markers, forming nodular vascular hyperplasia (12–14).

In this regard, anti-angiogenic therapy has emerged as a hotspot for drug research and development over the past three decades. Traditional monoclonal antibodies, represented by bevacizumab and cetuximab, have achieved significant clinical success owing to their high specificity and potent neutralizing capabilities. However, these agents exhibit several clinical limitations. Their large molecular weight (~150 kDa) severely restricts penetration into solid tumors. Additionally, Fragment crystallizable(Fc)-mediated effector functions and prolonged serum half-lives elevate the risks of off-target toxicity and immunogenicity (15). Furthermore, single-target blockade often induces compensatory bypass activation, culminating in acquired drug resistance (16). Consequently, developing novel anti-angiogenic therapeutics that combine high affinity, superior tissue penetrability, and low immunogenicity remains an urgent clinical priority.

Derived from camelid heavy-chain antibodies, nanobodies have emerged as promising candidates for next-generation targeted therapeutics due to their high solubility and modularity. Despite their compact size, they retain full antigen-binding capacity while exhibiting excellent tissue penetrability, rapid renal clearance, and robust stability. These features allow them to overcome the inherent limitations of traditional monoclonal antibodies, such as poor delivery efficiency and high immunogenicity, underscoring their strong potential for clinical translation (17–19). This review systematically examines the engineering strategies of anti-angiogenic nanobodies for translational applications. Furthermore, it highlights recent advances in humanization, multivalent and multispecific designs, targeted drug delivery, and theranostic platforms, concluding with a discussion of current challenges and future perspectives.

## 2 Structure and properties of nanobodies

**Figure 1:**
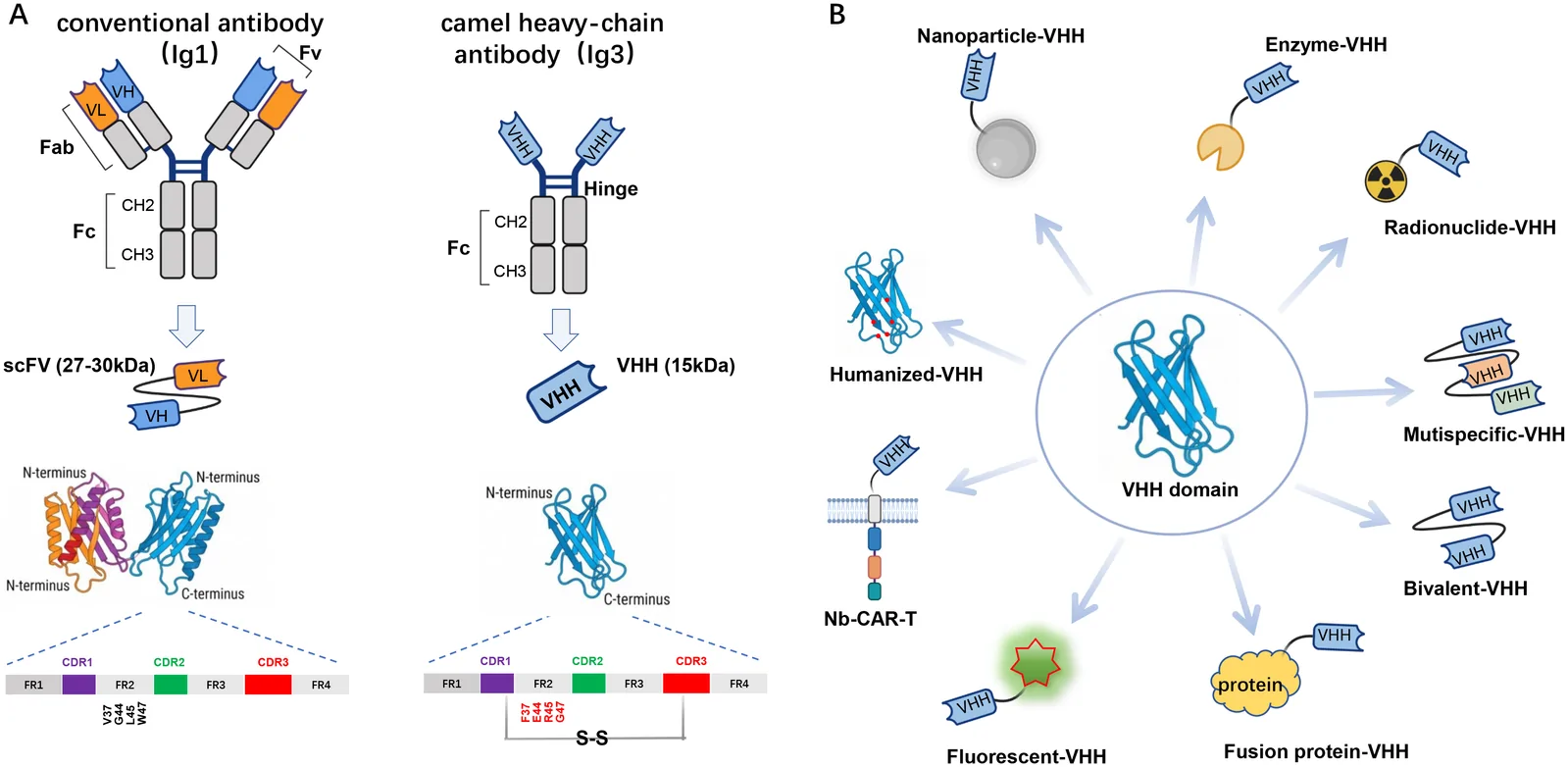
**(A)** Comparison of the monoclonal antibody (mAb) vs. heavy chain antibody (HcAb) to highlight the structural differences of their respective antigen binding regions. The VHH has a much longer CDR3 loop compared to that of the VH-VL domains in mAbs, providing antigen affinity and access to hidden epitopes. **(B)** A generalized overview of the types of engineered nanobodies to demonstrate how their high modularity enables various modifications.

In addition to the conventional heterotetrameric antibodies, camelids possess a unique class of serum antibodies known as heavy-chain antibodies (HCAbs), a type of Immunoglobulin G(IgG) that naturally lacks light chains. At its N-terminal region, the H chain of the homodimeric protein contains a dedicated variable domain, referred to as Variable Heavy-chain domain of Heavy-chain antibodies (VHH), which is the structural and functional equivalent of the fragment antigen-binding (Fab) of conventional antibodies (**Figure 1A**). VHH typically has a molecular weight of 12–15 kDa and dimensions of approximately 4 × 2.5 × 3 nm, meanwhile, the compact architecture of VHH enables the rational size engineering across a wide spectrum, ranging from a few nanometers for monomers to over 10 nm when formatted as multivalent constructs or conjugated to therapeutic payloads, largely dictated by the dimensions of the conjugated moieties (20). Structurally, VHH comprises four conserved framework regions (FR1–FR4) and three complementarity-determining regions (CDR1-3) (21). To compensate for the lack of a variable light chain (VL), the VHH CDR3 is significantly elongated (16–18 residues). This extended loop forms a protruding, “finger-like” structure capable of penetrating deep into antigen clefts or cryptic epitopes (22). Additionally, substituting key hydrophobic residues in the FR2 region with hydrophilic ones (e.g., F/Y42, E49, R50, and G52) markedly improves their solubility and prevents aggregation (23). Furthermore, an extra disulfide bond is often present, which enhances conformational stability and rigidity (24, 25). Consequently, VHH exhibits numerous advantages over full-length IgGs and traditional antibody fragments (e.g., scFv and Fab). Their high thermal stability, broad pH tolerance, high solubility, and low aggregation propensity facilitate long-term storage and administration. Moreover, their small size enhances penetration into dense tissues, such as solid tumors, while the absence of an Fc domain minimizes immunogenicity (26–28). VHHs with specific antigen-binding activity are isolated via library construction and display panning. Owing to their nanoscale size, these recombinant proteins are broadly known as nanobodies(Nbs) in downstream applications.

These characteristics of VHH endow Nbs with a highly modular structure, facilitating the implementation of various engineering strategies (**Figure 1B**). For instance, multivalent or bispecific constructs can be readily generated using flexible linkers (29, 30). Additionally, Nb functionality can be expanded by fusing them with effector molecules, such as drugs, therapeutic proteins, or imaging agents (17, 31). These modular designs greatly simplify the assembly of multifunctional molecules, facilitating their broad application in disease diagnosis, targeted therapy, and drug delivery. In summary, Nbs engineering is spearheading a new paradigm in cancer therapy while demonstrating profound potential for targeted interventions in other angiogenesis-dependent diseases.

## 3 Angiogenesis and immunological regulation in the tumor microenvironment

### 3.1 Angiogenesis mechanisms and molecular targets

**Figure 2:**
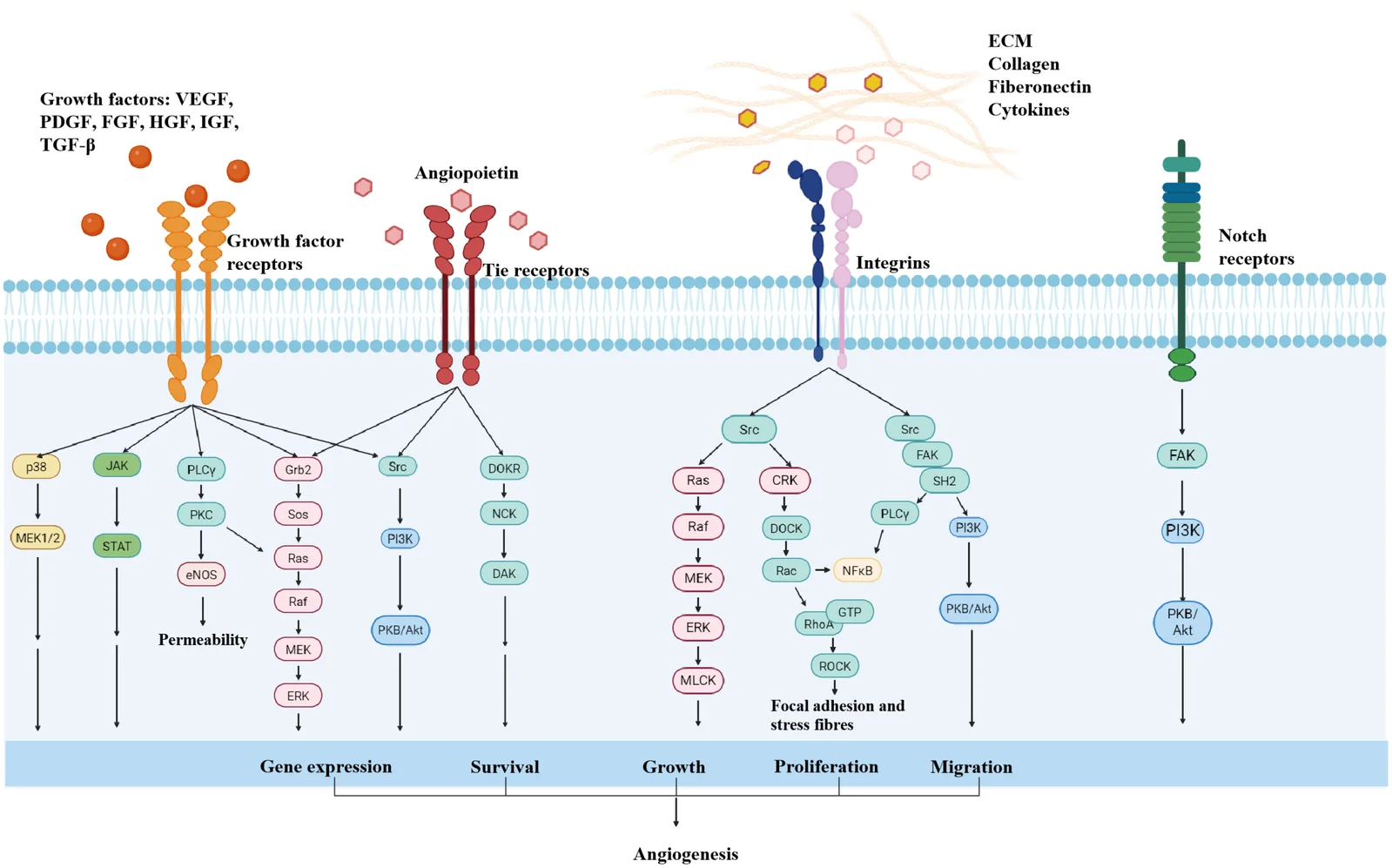
Schematic diagram of the signaling pathway network during angiogenesis. Solid arrows indicate activation; blunt arrows indicate inhibition.

The regulation of the angiogenesis involves a highly complex network of signaling pathways (2, 32, 33) (**Figure 2**). The VEGF/VEGFR axis acts as the primary driver of angiogenesis, activating downstream cascades such as PI3K/AKT and MAPK/extracellular signal-regulated kinase(ERK) to promote endothelial cell proliferation, migration, tube formation, and vascular permeability (34). Concurrently, the Angiopoietin-2/Tie2(Ang2/Tie2) and Notch/DLL4 pathways govern vascular maturation and maintain orderly branching (35). Additionally, the PDGF/PDGFR, FGF/FGFR, and HGF/c-Met pathways are crucial for pericyte recruitment, endothelial cell survival, and compensatory signaling following VEGF blockade (36). Furthermore, under hypoxic conditions, the HIF-1α-mediated transcriptional network upregulates various pro-angiogenic factors, acting as a critical microenvironmental regulator of this process (37).

Various biomolecules associated with these pathways—primarily pro-angiogenic factors, cell adhesion molecules, and microenvironmental regulators—serve as critical targets for nanobodies development (**Table 2**) (32). Among these, pro-angiogenic factors (e.g., VEGF, FGF, angiopoietin, HGF, and their receptors) remain the primary focus of current nanobodies research. As extracellular proteins or membrane-bound receptors, these targets are highly accessible for nanobodies binding, enabling the efficient blockade of downstream signaling cascades. Furthermore, nanobodies targeting cell adhesion molecules (e.g., integrins and cadherins) and microenvironmental regulators (e.g., matrix metalloproteinases and HIF-1α) offer dual clinical and diagnostic advantages. They can be co-administered with anti-angiogenic therapies to enhance efficacy and mitigate drug resistance, while simultaneously serving as novel molecular tools for real-time imaging and precise evaluation of angiogenesis (38, 39).

**Table 2:** Overview of nanobodies targeting angiogenesis-related factors by targets.

Nanobody target	Core biological mechanism	Model system	Outcomes	References
VEGF-A (VEGF165)	Binds to VEGFR-2 to activate PI3K/AKT and MAPK/ERK pathways, promoting endothelial cell (EC) proliferation, migration, tube formation, and vascular permeability.	Chick chorioallantoic membrane (CAM) assay; Human Umbilical Vein Endothelial Cells (HUVECs)proliferation/tube formation assay.	VA12 exhibits significant *in vivo* anti-angiogenic activity; the anti-VEGF nanobody dose-dependently inhibits HUVEC proliferation and tube formation.	(40, 41)
VEGFR-2	The primary receptor for VEGF, mediating EC proliferation, migration, survival, and tube formation.	HUVEC proliferation/tube formation assay; mouse pharmacokinetics (BALB/c).	Specifically binds to cell-surface VEGFR-2; almost completely inhibits HUVEC tube formation *in vitro*.	(42, 43)
Ang2 (Angiopoietin-2)	Binds to Tie2 to destabilize blood vessels, acting synergistically with VEGF to promote neovascularization; it is highly upregulated in tumors.	Phase Ib clinical trial (NCT03468426) enrolling patients with advanced solid tumors (e.g., Non-small cell lung cancer, metastatic Non-small cell lung cancer, Small cell lung cancer, melanoma, recurrent glioblastoma, hepatocellular carcinoma); mouse malignant glioma models (U87 MG, etc.).	BI 836880 monotherapy is 720 mg IV Q3W; exhibits manageable safety and preliminary antitumor activity when combined with BI 754091; Ang2/VEGF-A bispecific antibody monotherapy or in combination with chemotherapy demonstrates the most robust vascular normalization.	(44, 45)
HGF (Hepatocyte Growth Factor)	Binds to c-Met to activate the PI3K/AKT pathway, promoting EC survival, migration, and compensatory angiogenesis.	Nude mouse U87 MG glioblastoma xenograft model (biodistribution, 89Zr-Positron Emission Tomography(PET).	Blocks HGF/c-Met interaction, inhibiting cellular signaling and proliferation; demonstrates high tumor-targeting selectivity and prolonged blood exposure following ^89^Zr labeling; tumor growth is delayed or cured in the 100 μg dose group.	(46)
PLGF (Placental Growth Factor)	Binds to VEGFR-1, synergizing with VEGF to enhance angiogenesis and vascular permeability.	HUVEC MTT proliferation, 3D collagen tube formation, and MCF-7 migration assays; CAM model.	The bivalent nanobody binds to PLGF with high affinity; significantly inhibits HUVEC proliferation, 3D tube formation, and MCF-7 migration; angiogenesis is suppressed in the CAM model.	(47–49)
NRP-1 (Neuropilin-1, co-receptor)	Enhances VEGF-VEGFR-2 signaling, promoting EC migration and the recruitment of tumor-associated macrophages.	HUVEC MTT proliferation assay, Matrigel tube formation assay, CAM model, nude mouse HCT116 colon cancer xenograft model.	Enhances angiogenesis inhibition when combined with anti-VEGF therapy; significantly reduces tumor volume and weight in mouse models; high-affinity NRP-1 nanobody inhibits HUVEC proliferation, tube formation, and CAM angiogenesis.	(50–52)
DLL4 (Delta-like ligand 4)	A Notch ligand that regulates tip/stalk cell differentiation, preventing excessive vascular branching.	MKN cell MTT proliferation and Annexin V/PI apoptosis assays; chick CAM model.	DLL4 nanobody 3Nb3 binds to and is internalized by MKN cells; it dose-dependently inhibits proliferation and induces apoptosis; it induces abnormal/non-functional angiogenesis in the CAM model.	(53, 54)
PDGF-B	Binds to PDGFR-β, recruiting pericytes to cover newly formed blood vessels and maintaining vascular stability.	None		
CD105 (Endoglin, TGF-β co-receptor)	A specific marker for tumor neovascular ECs, promoting angiogenesis and pericyte recruitment.	HUVEC counting/MTT proliferation assay, fibrin gel tube formation assay.	Achieved high-affinity HUVEC-specific nanobody AR-86a; significantly inhibits HUVEC proliferation and capillary-like structure formation.	(55)
Integrin αvβ3/αvβ5	Mediates EC adhesion to the ECM, activates focal adhesion kinase/Src proto-oncogene tyrosine kinase signaling pathway, and promotes EC migration, survival, and vasculogenic mimicry.	MTT assay, SKOV3 xenograft mouse model.	The nanobody-drug conjugate exhibits superior targeting, cellular uptake, and cytotoxicity; demonstrates long-term *in vivo* retention and high tumor accumulation.	(39)
VE-cadherin (Vascular endothelial cadherin)	Maintains stable junctions between ECs, regulating vascular permeability and lumen assembly.	None		
CD93 (Endothelial cell surface receptor)	Participates in EC adhesion, vascular permeability regulation, and neovascularization; regulates the assembly of β1 integrin and fibronectin.	MTT assay, wound healing assay, Matrigel tube formation assay.	Anti-CD93 nanobodies NC81/NC89 significantly inhibit HUVEC proliferation, migration, and tube formation; they also downregulate VE-cadherin expression.	(56)
VCAM-1 (Vascular cell adhesion molecule-1)	Expressed on activated endothelium, promoting inflammatory cell recruitment and angiogenesis signal amplification.	Atherosclerotic double knockout mice, closed-loop model of human endarterectomy specimens; atherosclerotic mouse models, rabbit atherosclerotic models, human carotid/femoral endarterectomy specimens.	Microbubble signaling is enhanced in double knockout mice aortas and human specimens (detectable in early/late plaque inflammation); radiolabeled cAbVCAM-1–5 enables non-invasive imaging and quantification of the degree of atherosclerotic inflammation.	(38, 57, 58)
MMP-2/MMP-9 (Matrix metalloproteinases)	Degrades the basement membrane/ECM to release cryptic growth factors (e.g., VEGF, FGF), promoting EC migration.	Recombinant active human MMP-2, human cell lines: HEK293, HeLa, human washed platelets.	Inhibits MMP-2 gelatin hydrolysis; dose-dependently inhibits collagen-induced platelet aggregation, P-selectin expression, p38 phosphorylation, thrombin receptor activator peptide 6-enhanced aggregation, and platelet adhesion to MMP-2.	(59)
Fibronectin EDB (EDB splice variant)	A tumor-specific ECM component that supports neovascular stroma, EC attachment, and the release of pro-angiogenic signals.	Triple-negative breast cancer (TNBC) mouse model, pancreatic ductal adenocarcinoma (PDAC) mouse model, melanoma mouse model, breast cancer progression model (MMTV-PyMT mice), pulmonary fibrosis model.	^64^Cu-labeled NJB2 highly specifically detects primary tumors and micrometastases; capable of delivering imaging/therapeutic payloads to the ECM of disease sites.	(60, 61)
urokinase-type Plasminogen activator uPA/uPAR	Activates plasmin, synergizing with MMPs to degrade the ECM and providing a migration pathway for angiogenesis.	*In vitro* enzyme activity inhibition assay.	Specifically binds to uPA; capable of binding to uPA associated with active site inhibitors.	(62)
Tenascin-C	An ECM glycoprotein that regulates cell adhesion and migration, promoting tumor angiogenesis and stromal remodeling.	Ulcerative colitis colon tissue, oral squamous cell carcinoma (OSCC), gallbladder cancer liver metastasis.	Nb3/Nb4 restore the adhesion of tenascin-C(TNC)-impaired tumor cells/mesangial cells to the FN/TNC matrix; blocks TNC/CCL21-mediated chemotactic retention of DC2.4 dendritic cells.	(63)

### 3.2 Angiogenesis-driven immune evasion in the tumor microenvironment

Pathological angiogenesis intrinsically drives immune evasion within the tumor microenvironment (TME). Overexpression of pro-angiogenic factors, primarily VEGF, creates an immunosuppressive environment that hinders anti-tumor immunity (5). Specifically, VEGF impedes dendritic cell (DC) maturation and antigen presentation, blunting the initiation of adaptive immune responses. Furthermore, structurally chaotic and leaky tumor vessels generate local hypoxia. This hypoxic environment recruits and polarizes innate immune cells toward suppressive phenotypes, particularly M2-like tumor-associated macrophages (TAMs). These TAMs subsequently secrete additional pro-angiogenic factors, such as VEGF and matrix metalloproteinases (MMPs), establishing a positive feedback loop that exacerbates both vascular abnormalities and immunosuppression (64). Within the lymphoid compartment, pathological angiogenesis disrupts lymphocyte infiltration and effector functions. Tumor endothelial cells frequently downregulate critical adhesion molecules. This downregulation forms an “endothelial barrier” that restricts the extravasation of cytotoxic T lymphocytes (CTLs) and natural killer (NK) cells into the tumor core. Conversely, dysregulated vascular signaling and hypoxia preferentially facilitate the recruitment and survival of regulatory T cells (Tregs) and myeloid-derived suppressor cells (MDSCs). The accumulation of these suppressive populations further dampens CTL and NK cell activation, ultimately securing immune tolerance and driving tumor progression.

Nanobodies possess unique structural and pharmacological properties that advantageously remodel the immunosuppressive tumor microenvironment (TME) to enhance cancer immunotherapy. By precisely neutralizing critical pro-angiogenic axes (e.g., VEGF or Ang-2), anti-angiogenic nanobodies induce transient vascular normalization. This process restores the structural integrity of endothelial junctions, reduces vessel leakiness, and alleviates local hypoxia. Crucially, vascular normalization overcomes the restrictive endothelial barrier by upregulating essential adhesion molecules, such as ICAM-1 and VCAM-1. Consequently, this upregulation facilitates the robust infiltration of cytotoxic T lymphocytes (CTLs) and natural killer (NK) cells from the peripheral circulation into the tumor parenchyma (65).

### 3.3 Mechanisms of resistance to anti-angiogenic therapy

Resistance to anti-angiogenic therapy extends beyond the compensatory activation of alternative pro-angiogenic pathways. Tumors may preserve their vascular supply through vessel co-option, in which malignant cells infiltrate surrounding tissues and exploit pre-existing host vessels without inducing endothelial sprouting (66). Vasculogenic mimicry provides another VEGF-independent mechanism, whereby highly plastic tumor cells acquire endothelial-like properties and form perfusable vascular-like channels (67). Tumor-associated endothelial cells may also undergo phenotypic reprogramming, including endothelial-to-mesenchymal transition, leading to altered angiogenic receptor expression and reduced dependence on VEGF/VEGFR signaling (68). Collectively, these alternative vascularization mechanisms and cellular transitions can sustain tumor perfusion despite effective inhibition of classical angiogenesis.

Anti-angiogenic therapy may also exacerbate hypoxia and nutrient deprivation, thereby promoting metabolic adaptation through enhanced glycolysis, lactate utilization, mitochondrial reprogramming, and activation of cellular stress-response pathways (69). Treatment-induced hypoxia may further recruit tumor-associated macrophages, myeloid-derived suppressor cells, and other immunosuppressive populations that secrete alternative angiogenic mediators and suppress antitumor immunity (70). These resistance mechanisms may coexist and evolve dynamically during treatment, suggesting that durable vascular control is unlikely to be achieved through VEGF blockade alone. Their mechanistic diversity therefore supports the development of resistance-informed nanobody engineering strategies and mechanism-based combination therapies.

## 4 Engineering of angiogenesis-targeting nanobodies

Despite their inherent structural and biochemical advantages, nanobodies face several limitations in practical biomedical applications. First, their low molecular weight falls well below the glomerular filtration threshold (50–60 kDa), resulting in a short serum half-life (minutes to hours) that hampe7rs clinical translation (71). Second, monovalent nanobodies exhibit lower target affinity and lack Fc-mediated effector functions—such as antibody-dependent cellular cytotoxicity (ADCC) and complement-dependent cytotoxicity (CDC)—limiting their efficacy against complex diseases (72, 73). Concurrently, multispecific engineering strategies based on nanobodies are emerging as a pivotal approach to surmounting the bottlenecks in solid tumor therapy. By integrating dual-targeting modules against angiogenesis and immune checkpoints(ICs), these constructs facilitate mechanistic complementarity and synergy. Specifically, the anti-angiogenic component induces tumor vascular normalization, effectively rectifying aberrant pathological vasculature and reversing hypoxia-driven immunosuppression by attenuating the recruitment and activation of immunosuppressive cells—such as regulatory T cells (Tregs) and myeloid-derived suppressor cells (MDSCs)—mediated by hypoxia-inducible factor-1α (HIF-1α). Meanwhile, the ICs module directly targets inhibitory checkpoints (e.g., PD-1/PD-L1), reinvigorating the effector functions of exhausted T cells (Tex). This double strategy damages physical vascular barriers and blocks molecular checkpoint pathways, thereby efficiently converting immunologically “cold” tumors into “hot” ones. Finally, camelid-derived sequences retain a potential risk of triggering immune responses (74, 75). Therefore, engineered modifications are critical for translating these biological molecules into viable clinical therapeutics. This section details various modular engineering strategies and their therapeutic applications in angiogenesis-related diseases.

### 4.1 Humanization

VHH shares 80%–90% sequence homology with human antibody variable regions, thereby exhibiting lower immunogenicity compared to traditional heterologous antibodies. However, treatments for angiogenesis-dependent diseases, such as solid tumors and autoimmune disorders, typically necessitate chronic, repeated dosing over months or years. Chronic dosing regimens required for solid tumors or autoimmune disorders increase the risk of anti-drug antibody (ADA) development, as evidenced by clinical data showing ADA incidences exceeding 30% in some nanobody programs (ALX-0061) (76, 77). Consequently, humanization is essential to mitigate potential immunogenicity.

**Figure 3:**
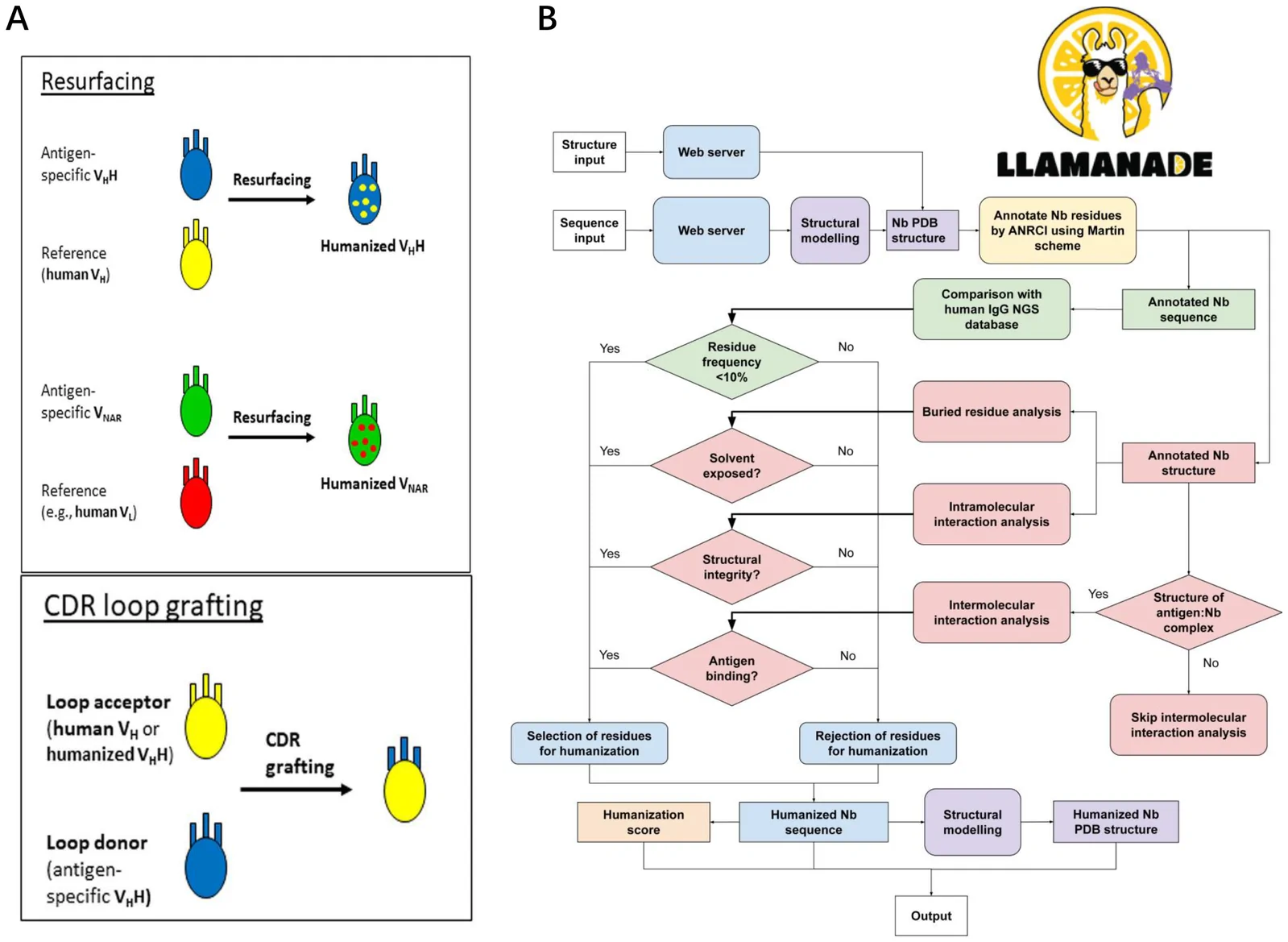
Nanobody humanization strategies and computational workflow. **(A)** Major strategies for nanobody humanization, including complementarity-determining region (CDR) grafting and resurfacing. Adapted with permission from Ref (86). Copyright (2026), John Wiley & Sons. **(B)** Schematic workflow of Llamanade. Adapted with permission from Ref (81). Copyright (2026), Elsevier.

Complementarity-determining region (CDR) grafting involves transferring VHH CDRs onto a human template framework (**Figure 3A**). For example, Vincke et al. (78) introduced a universal humanized nanobody framework, NbBCII10FGLA, which effectively preserves VHH stability and antigen-binding capacity and has been extensively validated. A more conservative strategy, known as resurfacing, involves analyzing VHH solvent accessibility to selectively replace only solvent-exposed framework residues while preserving buried residues and CDRs. This classic approach maximizes the retention of VHH solubility and CDR conformation. Kazemi et al. (79) applied this technique to replace nine surface residues on the VEGF-targeting nanobody Nb42. The engineered variant exhibited no significant structural deviations from wild-type Nb42, retained comparable ELISA binding and VEGF inhibitory functions, and demonstrated reduced immunogenicity based on T-cell epitope predictions. Building upon the universal NbBCII10FGLA template, Gonzalez et al. constructed a fully synthetic humanized VHH library. From this library, the screened NbH1 variant demonstrated improved solubility, thermal stability, and nanomolar affinity for VEGF. This study validates the utility of fully synthetic humanized libraries in discovering high-performance candidates for targeting angiogenic pathways (80). However, CDR grafting rely on the assumption that framework regions merely serve as inert scaffolds, this strategy overlooks the critical role of framework residues in VHH paratopes. Unlike conventional antibodies, VHHs might utilize residues in the FR2 region for antigen contact. Additionally, while resurfacing is presumed to retain solubility by leaving the non-human framework intact, the FR2 hallmarks that underpin nanobody solubility are themselves surface-exposed and non-human. Critically, validation in many studies is limited to ELISA-based affinity measurements, leaving unresolved whether humanizing mutations subtly disrupt conformational epitopes involving framework residues or compromise key physicochemical properties such as solubility and aggregation propensity.

Recently, the rapid accumulation of antibody sequence and structure data has established computationally assisted and AI-driven methods as core strategies for balancing sequence “humanness” and “naturalness” (77). Sang et al. (81) developed Llamanade, the first open-source humanization tool dedicated specifically to nanobodies. Utilizing large-scale sequence alignment, it identifies VHH-specific features and preferentially retains highly conserved FR2 residues and CDR3-associated sites, achieving precise control over framework resurfacing. Similarly, AbNatiV, developed by Ramon et al. (82), employs a Vector Quantized Variational Auto-Encoder (VQ-VAE) deep learning framework (**Figure 3B**). This tool accurately quantifies the naturalness and humanness parameters of sequences, directly guiding precise framework mutations, particularly at hallmark FR2 residues. As structure prediction models like AlphaFold increasingly integrate with humanization algorithms, future pipelines may directly generate clinical-grade, humanized nanobodies from target epitopes, significantly accelerating antibody drug discovery (83).

Although these computational tools mark a clear advance over traditional manual grafting, their validation approaches still suffer from notable limitations. Most studies rely primarily on in silico humanness scores (such as T20, H-score, Hu-mAb) and *in vitro* binding assays. As Gordon et al. (77) emphasize, the correlation between these humanness metrics and actual clinical immunogenicity remains weak (Pearson r≈0.21–0.34 for conventional antibodies, and even more limited for nanobodies). Moreover, the strong emphasis on maximizing “humanness” often creates trade-offs with other essential developability properties, particularly relevant for anti-angiogenic applications. For example, mutations introduced to reduce T-cell epitopes may unintentionally increase surface hydrophobicity or destabilize the interfaces needed for multivalent or bispecific constructs. While combining AlphaFold with humanization pipelines holds considerable promise for accelerating candidate generation, the field must move beyond single-parameter optimization. Future efforts should rigorously assess whether computationally humanized nanobodies preserve not only target affinity, but also the molecular stability and pharmacokinetic behavior required for effective tumor penetration and safe chronic administration.

A further limitation arises from bias in the datasets used to train and benchmark AI models. Public antibody repositories exhibit marked compositional skew: conventional human IgG sequences vastly outnumber camelid VHH entries, while available nanobody datasets underrepresent the diversity of camelid species, germline families, immunization protocols, antigen types, and experimental platforms. This bias carries direct biological consequences. Since VHH germline gene usage dictates CDR3 conformation, framework–paratope interactions, and biophysical stability, models trained on skewed repertoires may learn lineage-specific sequence biases. Consequently, performance often degrades on underrepresented VHH families or unconventional epitope topologies (84, 85). Such selection and survivorship biases risk constraining generative models to recapitulate historical discovery space rather than identifying novel, developable clinical candidates. To address these limitations, future AI pipelines should implement provenance-aware deduplication and enforce data splits based on germline classification and antigen clustering. Rigorous external validation is essential, utilizing unseen targets and underrepresented camelid lineages. This ensures model optimization reflects true translational utility rather than mere similarity to overrepresented training examples.

### 4.2 Multivalent/Multispecific assembly

Although monovalent nanobodies possess excellent tissue penetration and stability, their therapeutic utility is often hindered by low binding affinity, short serum half-lives, and an inability to simultaneously block synergistic signaling pathways, ultimately leading to drug resistance or insufficient efficacy (87, 88). Consequently, engineering nanobodies via multivalent and multispecific assembly has emerged as a key strategy to enhance their anti-angiogenic efficacy.

This approach primarily involves the tandem fusion of identical or distinct VHH domains using flexible or rigid linkers (e.g., (GGGGS)n or the llama IgG2c hinge region) to generate novel fusion proteins. For instance, a bivalent anti-VEGF nanobody constructed by Sadeghi et al. (89) significantly enhanced the inhibition of human umbilical vein endothelial cell (HUVEC) proliferation, tube formation, and migration, while extending the plasma half-life by approximately 1.8-fold. Huang et al. (90) further optimized the linker to construct a tetravalent anti-VEGF nanobody. This construct exhibited a 134-fold increase in binding affinity compared to its monovalent counterpart and an approximately 6-fold enhancement in blocking VEGF-VEGFR signaling. Furthermore, multispecific engineering via the modular fusion of distinct VHH domains allows for the simultaneous blockade of multiple signaling pathways, thereby overcoming the limitations of single-target therapy. This multispecific design helps prevent compensatory drug resistance typically triggered by single-target blockade. For example, tumors often achieve angiogenic compensation following VEGF blockade via the bypass activation of Ang-2/Tie2 or DLL4/Notch pathways. Co-targeting VEGF with either Ang-2 or DLL4 effectively neutralizes these compensatory mechanisms, thereby enhancing anti-angiogenic efficacy and reducing the risk of resistance (91, 92). Multispecific assembly also facilitates a synergistic effect between angiogenesis inhibition and immunomodulation. Within the tumor microenvironment (TME), abnormal angiogenesis and immune evasion are mutually reinforcing: neovascularization induces hypoxia and upregulates immunosuppressive factors, while immunosuppression exacerbates vascular abnormalities. To address this, Eskafi et al. (93) designed an anti-VEGF/anti-programmed death-ligand 1(PD-L1) bispecific nanobody. This molecule synergistically inhibited HUVEC and tumor cell proliferation and tube formation while concurrently blocking PD-L1-mediated T-cell suppression, thereby achieving dual anti-angiogenic and immune checkpoint blockade effects. Additionally, multispecific constructs are frequently employed to extend half-life by incorporating human serum albumin (HSA)-targeting domains, which prolong *in vivo* circulation time. For instance, BI 836880 (developed by Ablynx/Sanofi) fuses anti-VEGF, anti-Ang-2, and HSA-binding domains. Preclinical studies demonstrated that this molecule exhibits superior anti-proliferative and anti-angiogenic activities compared to single-target inhibition across multiple patient-derived xenograft models, boasting a plasma half-life of up to 11 days (94, 95).

However, these multivalent or multispecific constructs may carry certain inherent risks. Although fusing a HSA binding domain effectively mitigates the rapid renal clearance inherent to nanobodies, the resulting increase in molecular weight can erode the tissue-penetration advantage that otherwise favors nanobodies over conventional antibodies. Furthermore, the structural integrity of large assemblies such as BI 836880 is sensitive to pH and temperature; minor misfolding in one domain can expose hydrophobic patches, elevating aggregation and immunogenicity risks under chronic dosing regimens. These concerns are not merely theoretical. A systematic comparison of 64 TNF-targeting constructs demonstrated that bispecific and tandem-scFv formats consistently accrued more risks in aggregation, fragmentation, and interfacial stress assays than natural IgG, with scFv–scFv and diabody formats exhibiting the highest liability (96). The authors attributed fragmentation primarily to glycine–serine (GS) linkers, indicating that linker engineering (such as length tuning, disulfide stabilization) is an essential design step. Moreover, in silico tools which perform well for monovalent IgG or nanobody, often fail to resolve format-specific risks for these engineered constructs, echoing the AI-prediction problem raised in Section 4.1. On the other hand, many angiogenic targets are also present on normal blood vessels, blocking them can cause severe side effects. Although co-targeting VEGF and DLL4/Notch is mechanistically appealing for suppressing compensatory angiogenesis, clinical experience with demcizumab (a humanized anti-DLL4 IgG2) reported Grade III hypertension in 28% of patients, pulmonary arterial hypertension, and sinusoidal dilation with centrilobular hepatocyte atrophy, a class effect of DLL4/NOTCH1 blockade on the liver sinusoidal endothelium (97).

Recent studies have employed AI-driven design frameworks—integrating structure prediction algorithms such as AlphaFold2 and RFdiffusion—to elucidate the steric complementarity between antigen epitopes and VHH complementarity-determining regions (CDRs) (28). Nevertheless, as discussed in Section 4.1, these computational models face substantial hurdles: AlphaFold2 often fails to accurately predict the conformational dynamics of long linkers or the structural consequences of framework mutations introduced during humanization. Moreover, while AI can optimize in silico stability, it cannot yet reliably forecast *in vivo* pharmacokinetic interactions or the cumulative immunogenicity of multi-domain constructs. Therefore, while computational design can guide the initial drafting of multivalent Nbs, rigorous empirical validation remains irreplaceable for de-risking these complex modalities prior to clinical entry.

### 4.3 Nanobody-based functionalized drug carriers

Due to their lack of specificity, conventional chemotherapeutic drugs frequently induce severe systemic side effects, including myelosuppression, hepatorenal toxicity, and cardiotoxicity (98–100). Consequently, the targeted delivery of anti-tumor drugs via the specific recognition of angiogenesis-related targets has emerged as a prominent research focus. In these targeted systems, nanobodies serve as precision modules to confer cellular, stromal, or microenvironmental specificity to drug molecules. Currently, a variety of flexible strategies utilizing nanobodies for drug delivery have been developed, which can be broadly classified into nanobody-drug conjugates (NDCs) and nanobody-based drug delivery vehicles (NDVs).

#### 4.3.1 NDC

**Figure 4:**
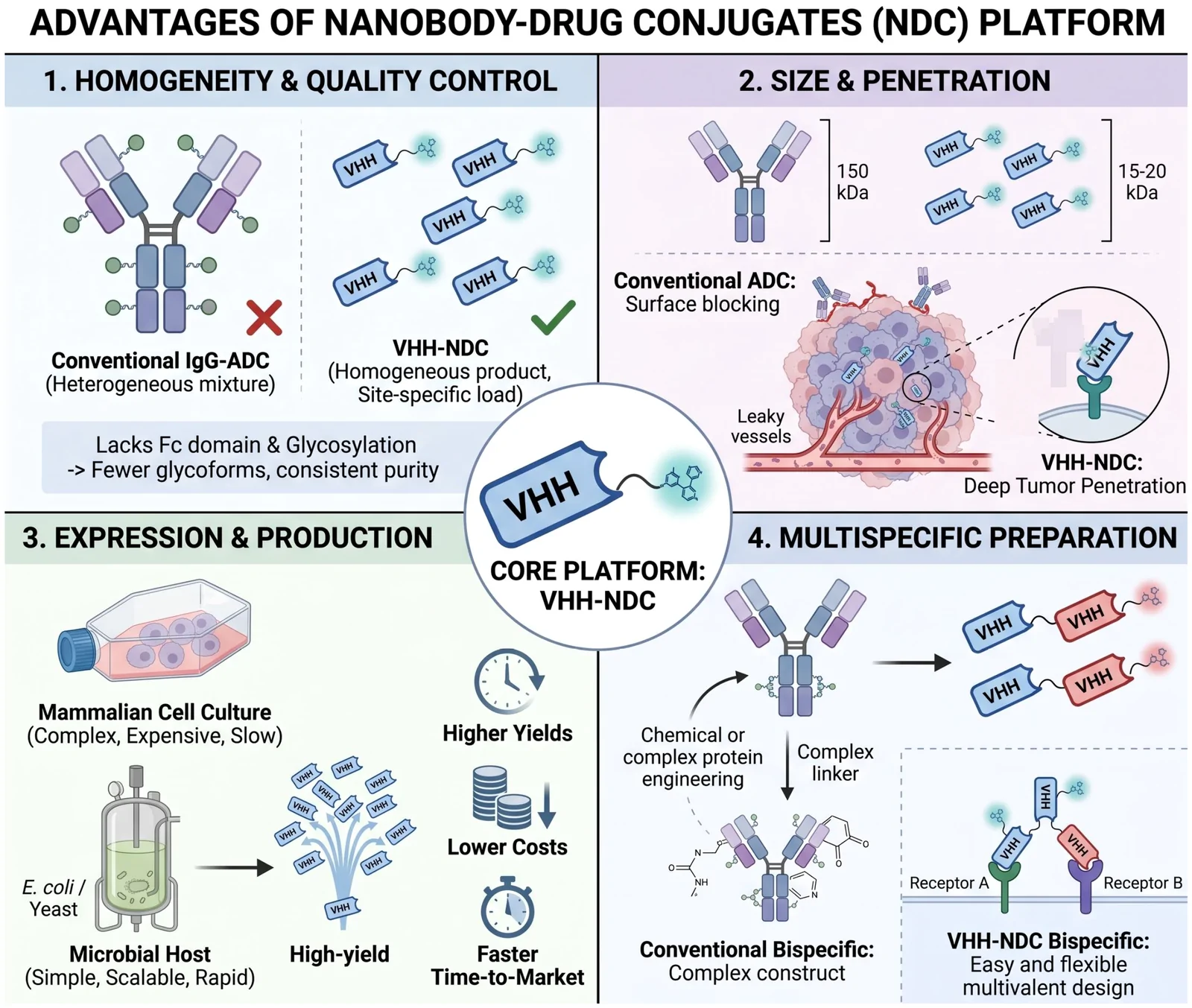
Unique advantages of nanobodies for drug conjugation. 1. Homogeneity and quality control: The absence of an Fc domain and glycosylation eliminates heterogeneity, ensuring uniform products and simplified quality control; 2. Enhanced tumor penetration: The small size (~15 kDa) facilitates deeper tissue penetration, particularly beneficial for accessing solid tumors; 3. Efficient production: microbial expression systems (e.g., *E. coli*) enable high-yield, low-cost, and rapid manufacturing compared to mammalian cell culture; 4. Multispecific design: The compact structure allows for facile generation of multivalent and multispecific conjugates.

Although antibody-drug conjugates have achieved significant clinical success, the large molecular weight of conventional monoclonal antibodies restricts their broader application (101–103). Consequently, NDCs are rapidly emerging as a promising alternative. Owing to their simple structure and low molecular weight, nanobodies possess unique advantages for drug conjugation (**Figure 4**). These advantages include the absence of an Fc domain and glycosylation, ensuring more homogeneous production and superior quality control (104). Additionally, their small size facilitates site-specific conjugation and enhances solid tumor penetration (105). Furthermore, their compatibility with microbial expression systems increases yield while reducing production costs and time (106). Finally, nanobodies offer greater ease in engineering multi-targeted conjugates (107).

**Figure 5:**
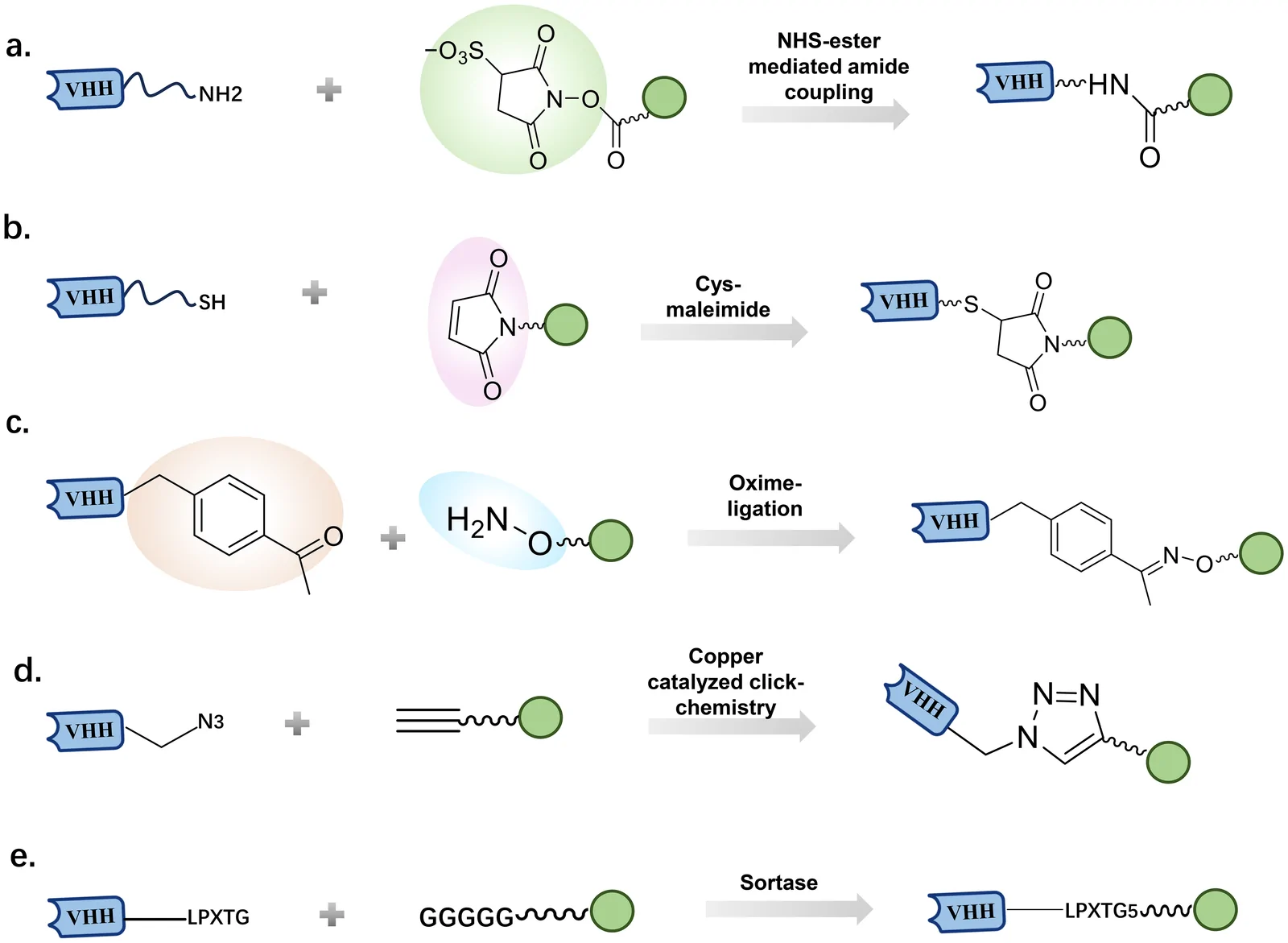
Common ways in which Nbs can be conjugated with drugs, where **(a)** represents random conjugation and **(b–e)** illustrate site-specific conjugation. **(a)** An activated carboxylic acid moiety reacts with a lysine residue, which results in amide bond linkage between Nb and the payload. **(b)** A maleimide moiety reacts with a cysteine residue of an Nb. **(c, d)** Non-natural amino acid incorporation by genetic engineering into Nbs and subsequent chemical conjugation (oxime ligation) and copper-catalyzed click-chemistry, respectively. **(e)** Site-specific (chemo) enzymatic sortase-mediated conjugation. Adapted with permission from Ref (108). Copyright (2026), Taylor & Francis.

NDCs consist of a nanobody linked to a therapeutic payload, with the conjugation strategy forming the core of their design. While protein-based payloads typically utilize gene fusion, small-molecule chemotherapeutics are covalently attached via chemical conjugation. This chemical linkage is broadly classified into random and site-specific conjugation (101, 108) (**Figure 5**). Random conjugation relies on the reaction of lysine amines or cysteine sulfhydryls with N-hydroxysuccinimide (NHS) esters or maleimides. Although straightforward, this method often yields a heterogeneous drug-to-antibody ratio (DAR). Moreover, modifying lysine residues within the complementarity-determining regions (CDRs) can compromise target binding activity. Consequently, site-specific conjugation has emerged as the preferred strategy. Common approaches include C-terminal engineered cysteine conjugation (109), sortase A-mediated conjugation (110), and unnatural amino acid incorporation technologies (111, 112). Although these techniques present specific challenges—such as reduced expression yields (113), low overall conjugation efficiency (114), and the potential introduction of immunogenicity (115), they enable selective modification of specific amino acid sequences, allowing for precise control over the DAR.

In 2013, Behdani et al. (116) pioneered the design of angiogenesis-targeting nanobody-drug conjugates (NDCs). They screened and isolated the specific anti-VEGFR2 nanobody 3VGR19, which they then fused with a truncated *Pseudomonas aeruginosa* exotoxin A. *In vitro*, this complex selectively inhibited VEGFR2-positive cell proliferation, confirming its anti-angiogenic and anti-tumor potential. Subsequently, the team chemically conjugated the anti-VEGFR2 nanobody to a diphtheria toxin fragment via a sulfo-SMCC linker (117). The resulting VGRNb-DT immunotoxin demonstrated favorable therapeutic efficacy in the prostate cancer PC-3 cell line. Notwithstanding these promising preclinical advances, the therapeutic translation of NDCs is hampered by a critical pharmacokinetic liability inherent to their minimal architecture: rapid renal clearance and metabolic instability (118). While strategies such as PEGylation have been employed to prolong systemic exposure of the NDCs (119), they simultaneously increase hydrodynamic volume. This creates a pharmacodynamic paradox where enhanced circulatory retention comes at the expense of tumor penetrability. Striking the right balance between circulatory stability and tissue accessibility will be essential for advancing NDCs into successful clinical applications.

#### 4.3.2 NDV

Nanobody-based drug delivery vehicles (NDVs) represent another critical direction in nanobody engineering. Unlike directly conjugated NDCs, NDVs utilize nanobodies as targeting ligands to decorate the surfaces of carriers such as liposomes, polymeric micelles, dendrimers, or inorganic nanoparticles. Solid tumors typically exhibit hyperpermeable vasculature and lack functional lymphatic drainage, leading to the well-established enhanced permeability and retention (EPR) effect that permits the preferential accumulation of macromolecules and nanoparticles (120, 121). NDVs capitalize on this pathophysiological feature by combining the EPR effect with active targeting mediated by surface-displayed nanobodies, these systems achieve high drug loading, prolonged circulation, and specific extravasation into the tumor vasculature or neovascular microenvironment. Notably, angiogenesis-related targets, such as VEGF/VEGFR and EGFR, are highly active in NDV research and have been successfully utilized for the surface modification of various drug carriers (17, 20, 108).

**Figure 6:**
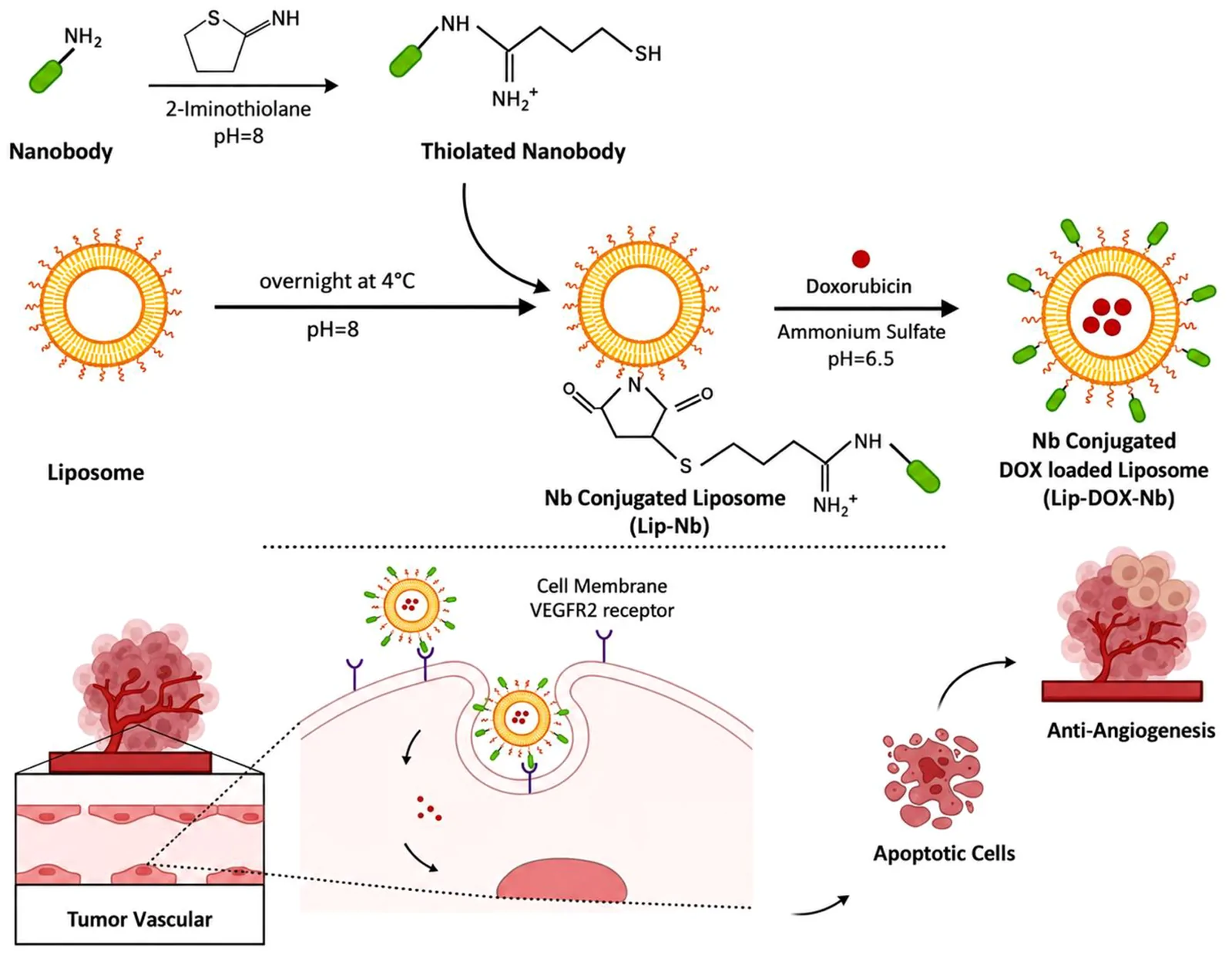
Schematic representation of the Nanobody-conjugated DOX Loaded liposomes preparation and interaction of this DDS with tumoral ECs. As proposed, the DOX-containing liposomes attach to tumoral ECs by VEGFR2 Nanobodies as targeting moieties and cause apoptotic and anti-angiogenesis effects. Reproduced from Akbari et al. (122), licensed under CC BY-NC 4.0.

Liposomes, which consist of spherical vesicles comprising one or more concentric lipid bilayers surrounding an aqueous core, are among the most well-established carriers utilized in NDVs (108). Akbari et al. (122) employed an anti-VEGFR2 nanobody to modify doxorubicin-encapsulating liposomes, constructing the Lip-DOX-Nb liposomal delivery system. Flow cytometry demonstrated that HUVECs exhibited a significantly enhanced uptake of these anti-VEGFR2-conjugated liposomes. Furthermore, Lip-DOX-Nb liposomes exerted stronger cytotoxic effects on VEGFR2-positive HUVECs, induced HUVEC apoptosis, and attenuated the migration of U87 cancer cells (**Figure 6**). Dendrimers are spherical polymeric nanoparticles composed of radially symmetric, branched monomers arranged layer-by-layer (108). Wu et al. (123) modified PEGylated dendritic nanoparticles with an anti-EGFR nanobody (7D12) to deliver a tetravalent platinum(IV) prodrug. Due to its exceptionally large size, this delivery system possesses a prolonged half-life and increased tumor drug accumulation. Consequently, it exhibited significantly enhanced anti-tumor efficacy in a nude mouse model bearing A431 (EGFR+) tumor xenografts. This study highlighted the robust potential of nanobody-conjugated, dendrimer-based delivery systems for targeted cancer therapy. Wu et al. (124) developed an enzyme-mediated, site-specific PEGylation strategy utilizing microbial transglutaminase (TGase) as a catalyst. By introducing a specific glutamine tag onto the nanobody, TGase catalyzed the transamidation reaction between PEG-NH_2_ and the glutamine residue, achieving precise site-specific conjugation. Building on this strategy, they constructed a nanobody(PEGylated)/Doxorubicin/HSA-UCNP (upconversion nanoparticle) composite nano-delivery system using an anti-epidermal growth factor receptor (EGFR) nanobody (7D12) as the targeting molecule. This complex exhibited significant doxorubicin (DOX) accumulation and cytotoxicity in EGFR-positive A431 cells. Conversely, it showed minimal effects in EGFR-negative MCF-7 cells, demonstrating robust targeting specificity. Additionally, specific nanobodies have been successfully applied to the surface modification of polymeric micelles (125, 126), albumin nanoparticles (127), silica nanoparticles (128), and gold nanoparticles (129). Furthermore, the aforementioned stimuli-responsive release technologies can be integrated into NDV construction. As classic biocompatible materials, these carriers readily accommodate stimuli-responsive designs, achieving controlled drug release specifically within targeted angiogenic regions (130). While the diversity of NDV platforms is impressive, the field faces significant scrutiny regarding clinical translatability. Preclinical efficacy data are overwhelmingly derived from subcutaneous xenografts, models known to overstate the Enhanced Permeability and Retention (EPR) effect relative to spontaneous orthotopic or patient-derived tumors. Furthermore, long-term biosafety profiles are lacking, particularly regarding carrier biodegradation and biocompatibility. Inorganic nanoparticles, such as gold and silica, present a specific risk of chronic sequestration within the reticuloendothelial system, raising concerns about cumulative organ toxicity.

Although most NDC and NDV studies have demonstrated encouraging efficacy in both *in vitro* and *in vivo* tumor models, the supporting data are largely restricted to early preclinical research and lack long-term safety and stability investigations (17). Additionally, current delivery systems targeting angiogenesis focus primarily on oncological indications, whereas their application in other angiogenesis-dependent diseases, such as wet age-related macular degeneration (wAMD), remains in the early exploratory stages. For example, one study reported that a bevacizumab-modified multivesicular liposome system (Bev-MVLs) effectively inhibited the formation and leakage of choroidal neovascularization (CNV). This finding highlights the significant potential of these systems for the localized, long-acting treatment of ophthalmic neovascular diseases (131). However, nanobody delivery systems targeting the same antigen have not yet been explored for ocular therapies.

Recently, the development of carrier-free or carrier-minimized systems has gained significant momentum in the field of nanodrug delivery. These designs utilize the self-assembly capabilities of specific drug molecules or conjugates to form nanoparticles without traditional carriers. Consequently, they maximize drug-loading capacity per unit volume while maintaining precise targeting, stable pharmacokinetics, and therapeutic efficacy (132–134). Given the inherent attributes of nanobodies—such as small molecular weight, modularity, and high target specificity—novel carrier-free or carrier-minimized strategies could be applied to develop self-assembling nanobody-drug systems. For instance, a 2026 study detailed a HER2-targeted mRNA- lipid nanoparticle(LNP) vaccine platform based on the self-assembly of palmitoylated nanobodies (pNBs) (135). Through electrostatic adsorption and hydrophobic interactions, the researchers co-assembled pNBs, lipids, and SP-encoding mRNA into LNPs. This approach successfully yielded a PEG-free LNP platform with high-density targeting capabilities and a controllable structure. Importantly, as preclinical research on NDCs and NDVs is well-established, the underlying theoretical frameworks—including pharmacokinetic profiles and *in vivo* targeting mechanisms—provide a solid foundation for the rational design of next-generation delivery systems.

### 4.4 Nanobody-based molecular probes

Pathological angiogenesis involves complex spatiotemporal dynamics throughout its onset, progression, and response to therapy. Compared to traditional morphological imaging modalities (e.g., Optical Coherence Tomography(OCT) and fluorescence angiography), molecular imaging enables the non-invasive, real-time, and quantitative visualization of angiogenesis-related molecular and cellular events *in vivo*. This capability drives a paradigm shift in disease management toward early molecular recognition, precise subtyping, dynamic efficacy monitoring, and theranostics (136, 137). Concurrently, owing to their unique pharmacokinetic profiles, nanobodies have been widely applied across various molecular imaging modalities. This has led to the development of engineered strategies tailored for immuno-Positron Emission Tomography (PET), immuno-Single-Photon Emission Computed Tomography (SPECT), and near-infrared fluorescence (NIRF) imaging.

This section highlights the application strategies and prospects of nanobodies in nuclear medicine imaging (PET/SPECT), NIRF optical imaging, ultrasound microbubble contrast imaging, and magnetic resonance imaging (MRI) (138, 139). Following engineering modifications, nanobodies can be site-specifically or randomly conjugated with radionuclides (as illustrated in **Figure 5**). Positron-emitting nuclides (e.g., ^18^F, ^68^Ga, and ^89^Zr) are typically used for PET imaging (139), whereas gamma-emitting nuclides (e.g., ^99m^Tc, iodine-131 (^131^I), and lutetium-177 (^177^Lu)) are employed for SPECT imaging (140). Early studies successfully conjugated EGFR-targeting nanobodies with ^68^Ga (^68^Ga-7D12) and ^99m^Tc-tricarbonyl (^99m^Tc-8B6 and ^99m^Tc-7C12). In tumor-bearing nude mice, these tracers exhibited high tumor uptake, low hepatic retention, and rapid blood clearance, making them ideal probes for early, non-invasive radioimmunoassays (141–143). Beyond imaging tumor vasculature, researchers have radiolabeled nanobodies targeting the macrophage mannose receptor (MMR) and vascular cell adhesion molecule-1 (VCAM-1) with ^99m^Tc. These have been utilized for SPECT/micro-CT imaging in animal models of rheumatoid arthritis and atherosclerosis, respectively (138, 140, 144). Findings confirmed that these molecular probes can sensitively and reproducibly monitor disease-specific pathological changes. Notably, several preclinical achievements have rapidly progressed to clinical validation (145, 146). For instance, the VCAM-1-targeting ^99m^Tc-cAbVCAM1–5 immuno-SPECT tracer has entered a Phase I clinical trial (NCT04483167) to assess the inflammatory burden of atherosclerosis (147). Concurrently, an MMR-targeting nanobody tracer, [^68^Ga]Ga-NOTA-anti-MMR-VHH2, is undergoing Phase I/II clinical trials (NCT04168528 and NCT04758650) for atherosclerotic and tumor lesions (148, 149). Thus, these engineered constructs not only validate the feasibility of targeting angiogenesis and the associated inflammatory microenvironment but also serve as promising molecular imaging tools with clinical translational potential for the early diagnosis and therapeutic monitoring of angiogenesis-dependent diseases.

Near-infrared fluorescence (NIRF) imaging is another commonly utilized *in vivo* imaging modality. Probes constructed with near-infrared fluorophores offer significant advantages, including a high signal-to-noise ratio and deep tissue penetration. Conjugating fluorophores to antibodies that target tumor angiogenesis generates probes capable of providing real-time intraoperative navigation during solid tumor resection, pioneering a novel direction for fluorescence image-guided surgery (31). Among these, tracers resulting from the conjugation of the near-infrared dye IRDye800CW with traditional monoclonal antibodies, such as cetuximab (anti-EGFR) and bevacizumab (anti-VEGF), have already entered clinical trials (150, 151). Given their rapid blood clearance and capacity for deep tissue penetration, nanobodies are poised to become an ideal choice for NIRF imaging probes. An early study conjugated the IRDye800CW fluorophore to an EGFR-targeting nanobody, successfully achieving precise intraoperative localization of orthotopic tongue tumors and cervical lymph node metastases in a mouse model (152). Debie et al. (153) employed an IRDye800CW-conjugated anti-HER2 nanobody for imaging in a HER2-positive breast cancer model. This probe clearly visualized sub-millimeter lesions and accumulated in tumors 20 times faster than trastuzumab, thereby exhibiting superior tumor-to-background contrast. A recent study reported an αvβ3 integrin-targeted theranostic nanobody-drug prodrug, which utilized a glutathione-responsive disulfide linker to achieve the selective release of doxorubicin within the tumor microenvironment; simultaneously, the incorporated aza-BODIPY near-infrared fluorophore provided an activatable imaging function, enabling precise monitoring of prodrug activation and biodistribution (39). While elegant in design, this construct currently represents emerging hypotheses requiring rigorous validation. The incorporation of activatable fluorophores and payloads adds layers of complexity regarding *in vivo* stability, bioavailability, and predictable activation kinetics within the heterogeneous tumor microenvironment. Additionally, the increased molecular size from payload conjugation risks compromising the intrinsic tissue penetration advantage of naked nanobodies.

Furthermore, ultrasound, MRI, and quantum dot (QD) technologies can be integrated with nanobodies to develop novel molecular imaging modalities. Ultrasound molecular imaging requires microbubbles, which are microspheres filled with inert fluorocarbon gases. Microbubbles conjugated with an anti-VCAM-1 nanobody (µB-cAbVCAM1-5) have been successfully employed for ultrasound imaging in tumor-bearing mice (154). In a subsequent study, Mukesh et al. (57) designed another anti-VCAM-1 nanobody-based microbubble (MBcAbVcam1-5) and demonstrated its potential for clinical translation using mouse atherosclerosis models and ex vivo human specimens. Nanobody-coated superparamagnetic nanoparticles and magnetoliposomes have also been successfully utilized for magnetic resonance imaging (MRI) of tumors targeting various other antigens. Quantum dots (QDs) are spherical, photoluminescent semiconductor nanocrystals with potential for tumor imaging. Wang et al. (125) employed quantum dots as drug carriers and coated them with an anti-EGFR nanobody for theranostic applications. However, the application of such constructs remains limited by their poor biocompatibility (155).

**Figure 7:**
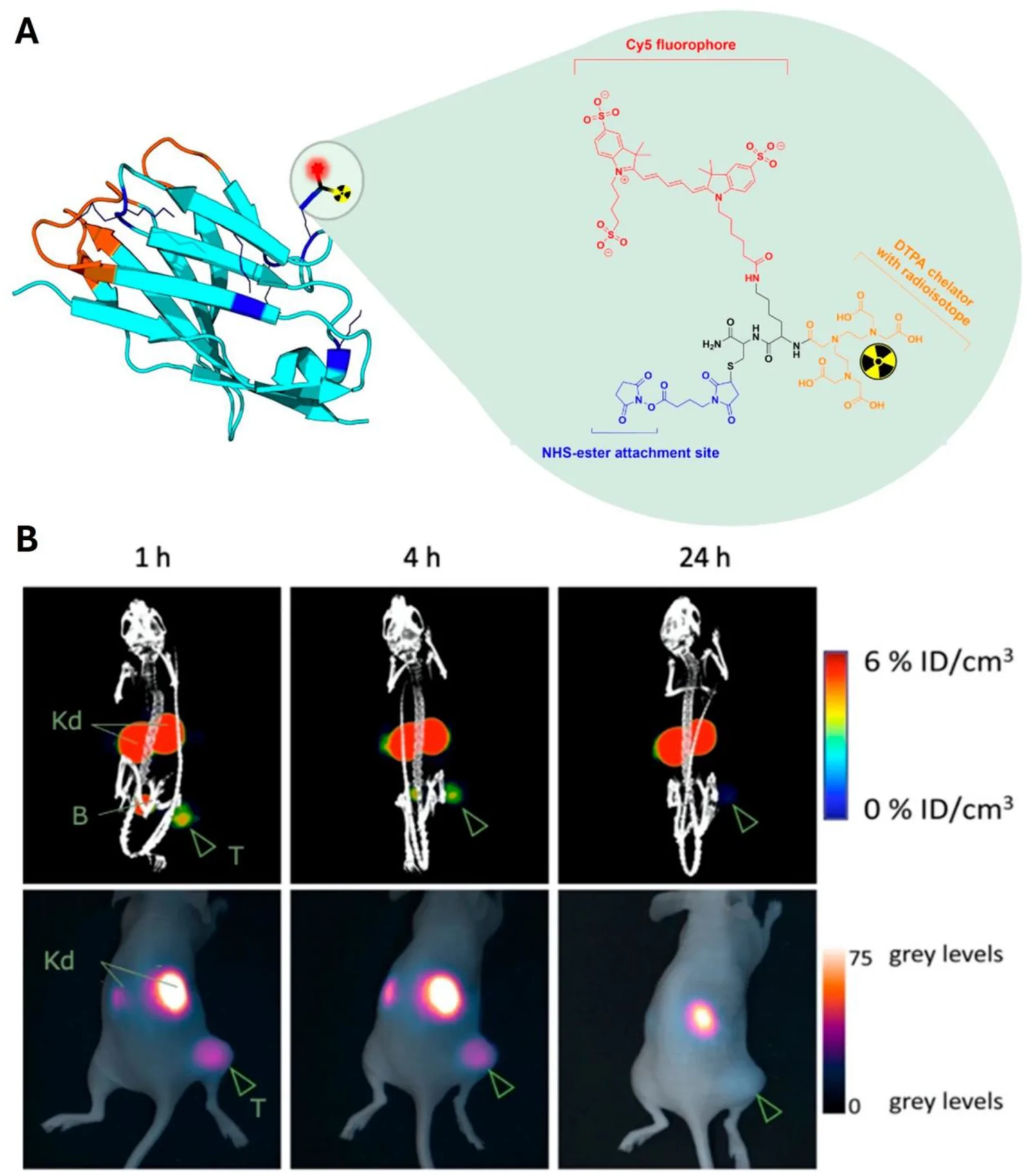
Concept and preclinical performance of bimodal nanobody-imaging agent [^111^In]In-MSAP.2Rs15d. **(A)** Schematic representation of the anti-HER2 nanobody 2Rs15d being randomly labeled on the lysines via NHS-activated ester bioconjugation chemistry (blue) with the MSAP (multifunctional single attachment point) containing a Cy5 dye (red) and a DTPA-chelator complexed with an indium-111 (111In) radioisotope (yellow). **(B)** Representative SPECT/CT (top) and fluorescence (bottom) images of an ovarian (SKOV3) tumor-bearing mouse at 1 h, 4 h, and 24 h post-injection of [111In]In-MSAP.2Rs15d. Specific uptake can be observed in the tumor (T), with little to no non-specific uptake, except for in the kidneys (Kd) and the bladder (B). This results in the specific and high-contrast imaging of HER2-positive subcutaneous tumors. Reproduced from Mammadbayli et al. (160), licensed under CC BY 4.0.

Recently, nanobody-based imaging research has transitioned from standalone diagnostics to theranostics (136, 139). For instance, radionuclides such as ^131^I (iodine-131) and ^177^Lu (lutetium-177) emit γ-rays suitable for SPECT imaging and also serve as medium-energy β^−^ particle emitters. When conjugated with nanobodies, these isotopes facilitate targeted radiotherapy for micrometastases and residual lesions (156). In photodynamic therapy (PDT), nanobodies are frequently employed as targeting modules conjugated with near-infrared emissive photosensitizers. This strategy not only leverages NIRF imaging to delineate tumor infiltration margins precisely but also enables laser-induced reactive oxygen species (ROS) generation, thereby triggering tumor cell apoptosis or necrosis (157–159). Furthermore, dual-modality nanobody tracers integrating NIRF and nuclear medicine imaging have emerged as a significant research focus. This design achieves spatiotemporal complementarity (**Figure 7**): the deep-tissue sensitivity of radionuclide imaging facilitates preoperative planning, while intraoperative fluorescence imaging provides real-time visual guidance (160, 161).

A fundamental biological barrier to molecular probe to clinical success is tumor and vascular heterogeneity. Angiogenesis-related targets (e.g., VEGFR2, EGFR, αvβ3) are often expressed inconsistently across patients and even within a single tumor. A single-targeted probe may yield a false negative if the target is downregulated in a specific lesion or during therapy. This has spurred interest in multi-specific probes (162, 163). However, these constructs face severe developability challenges: increased molecular weight and hydrodynamic radius directly impair renal clearance, potentially exacerbating off-target toxicity (e.g., renal retention of radioisotopes) and negating the advantage of VHHs. From a rational design perspective, future strategies might prioritize pairing a broad-spectrum angiogenic marker (e.g., VEGFR2) with a heterogeneity-specific or immune-checkpoint marker (e.g., PD-L1) (163). The goal is to maximize information gain while minimizing structural redundancy relative to indiscriminate multi-arm assembly.

### 4.5 Nb-CAR-T

Chimeric Antigen Receptor T-Cell (CAR-T) therapy has achieved remarkable clinical efficacy in hematological malignancies. However, its application in solid tumors remains limited by immunosuppression within the tumor microenvironment (TME) and insufficient T-cell infiltration. Traditional CAR architectures based on single-chain variable fragments (scFvs) are prone to misfolding or aggregation, which triggers ligand-independent tonic signaling and premature T-cell exhaustion. In contrast, nanobody-based CARs are monomeric and highly soluble, facilitating reliable folding and reducing the propensity for aggregation. This reduces basal signaling and enhances the functional persistence of engineered T cells (164, 165). However, these advantages are not universal, nanobody performance still depends heavily on specific clone selection, affinity, and CAR architecture, and extensive side-by-side screening is often required to identify optimal candidates.

Recent studies indicate that Nb-CAR-T cells exhibit exceptional anti-tumor activity when targeting the tumor vascular microenvironment. In 2019, Taheri et al. developed second-generation VHH-based CAR-T cells targeting VEGFR2. These cells specifically lysed VEGFR2-positive vascular endothelial cells while inducing T-cell activation and the secretion of interleukin-2(IL-2) and IFN-γ (166). In a 2024 study, the team optimized this design by adjusting the spacer length to construct a novel generation of Nb-CAR-T cells, which demonstrated enhanced cytokine release and increased cytotoxicity against VEGFR2-positive targets (167). Notably, VEGFR2 is constitutively expressed on healthy vascular endothelium, consequently, CAR-T therapies targeting this antigen are prone to dose-limiting toxicities (168). Given their high specific expression on tumor neovascular endothelial cells, CD105 (Endoglin) and the EIIIB (ED-B) fibronectin variant are ideal targets for Nb-CAR-T cells designed to disrupt the vascular microenvironment (169). Mo et al. utilized CRISPR/Cas9 technology to perform site-specific gene knock-in at the adeno-associated virus integration site 1 locus, successfully obtaining a CAR-T cell line with stable CAR expression; subsequent *in vivo* and *in vitro* experiments confirmed that targeting CD105 could concurrently lyse both tumor cells and tumor neovascular endothelial cells (170). Furthermore, Nb-CAR-T cells targeting the EIIIB (ED-B) fibronectin variant exert potent anti-tumor effects via direct cytotoxicity and nutrient deprivation (171). Critical gaps persist, however. While CD105 is upregulated on endothelial cells during physiological processes such as wound healing and placental development, its targeting poses a tangible risk of on-target, off-tumor toxicity in non-malignant contexts. At the same time, although ED-B offers a more tumor-restricted profile, its heterogeneous expression across histologies and paucity in several vascular-rich malignancies constrain its broad clinical utility. Compounding these issues, the current preclinical efficacy data derive predominantly from subcutaneous cell-line-derived xenografts (CDXs). These models fail to recapitulate the hallmark desmoplastic stroma and immunosuppressive microenvironment of human solid tumors which are frequently underacknowledged in early studies.

In recent years, the modular nature of nanobodies has continued to expand the boundaries of CAR-T engineering design. Tandem CARs (TanCARs) constructed through multivalent targeting designs effectively overcome tumor antigen heterogeneity and immune escape; simultaneously, the modularity of nanobodies has driven the development of logic-gated and tunable CAR systems, significantly enhancing the safety and targeting precision of the therapy (164, 172, 173). In summary, the unique physicochemical properties and engineering versatility of VHH position Nb-CAR-T cells as a promising platform for solid tumor therapy, particularly for vascular disruption strategies. However, realizing this potential will require addressing persistent translational hurdles through comparative studies of CAR-T engineering design, mechanistic insights into differential outcomes, and cautious progression from preclinical models to well-controlled clinical trials.

### 4.6 Integrated engineering of angiogenesis-targeting nanobodies

**Figure 8:**
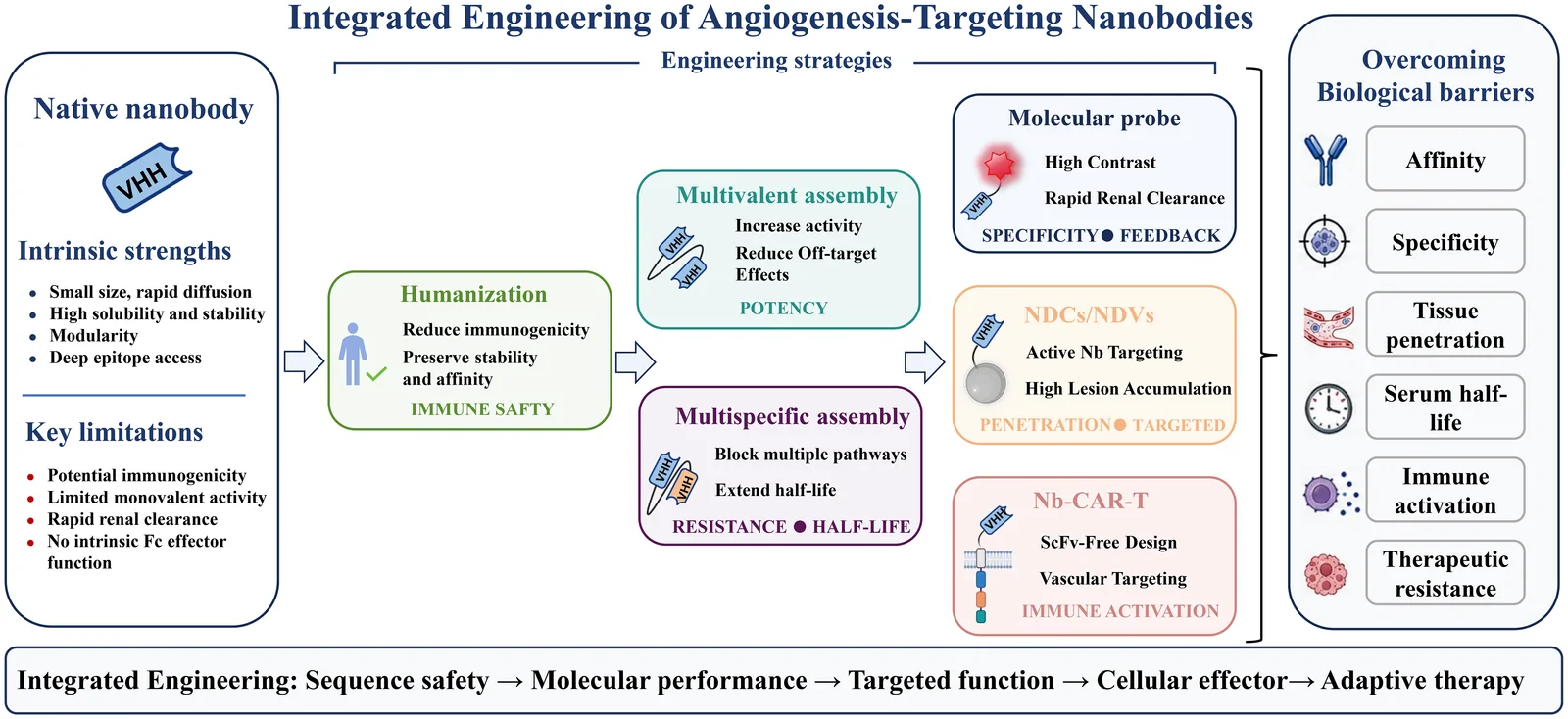
Integrated mechanistic framework for angiogenesis-targeting nanobody engineering. The VHH provides small size, rapid diffusion, solubility, and modularity, but is constrained by potential immunogenicity, limited monovalent affinity, rapid renal clearance, and the absence of intrinsic Fc effector function. Humanization, multivalent assembly, multispecific engineering, NDCs or NDVs, molecular imaging, and Nb-CAR-T technologies form complementary engineering layers that collectively address affinity, specificity, tissue penetration, serum half-life, immune activation, and therapeutic resistance. The framework also emphasizes imaging-guided feedback and the cross-cutting trade-off between increasing molecular size or complexity and preserving penetration, stability, and manufacturability.

The engineering strategies discussed above are not functionally isolated, because each modification influences multiple interdependent properties, and each biological barrier often requires a combination of approaches (**Figure 8**). Humanization primarily reduces the risk of anti-drug immune responses during repeated administration, while also providing a suitable sequence and structural basis for subsequent multivalent fusion, multispecific assembly, conjugation, and cellular engineering. Multivalent assembly increases functional affinity through avidity and may enhance target retention and systemic exposure (89). Albumin-binding domains and other half-life-extension modules further reduce rapid renal clearance (94). However, these modifications increase molecular size and may partially diminish the tissue-penetration advantage of monomeric nanobodies. Affinity, serum half-life, and tissue penetration must therefore be optimized collectively. Compact monomers may be preferable for rapid imaging, local administration, or access to poorly perfused lesions, whereas multivalent or half-life-extended formats may be better suited to sustained systemic inhibition of angiogenic signaling.

Multivalency, multispecificity, drug delivery, and CAR-T engineering represent overlapping levels of functional enhancement: multivalency strengthens antigen engagement, multispecificity broadens pathway coverage, conjugation introduces a pharmacological effector, and CAR-T technology provides an amplifiable cellular immune response (87–95, 101–108, 164–170). Their coordinated application may improve target engagement, address antigen and pathway heterogeneity, and reduce therapeutic resistance. Radiolabeled or fluorescent nanobodies can determine whether a candidate target is sufficiently expressed and accessible, whether compact or half-life-extended formats reach the intended tissue compartment, and whether target heterogeneity is likely to limit monospecific therapy. These data can guide the selection of multispecific constructs, nanobody–drug conjugates, delivery vehicles, or Nb-CAR-T cells according to the spatial distribution and biological context of the target (39, 152, 153). Longitudinal imaging may also reveal target loss, incomplete tissue penetration, vascular remodeling, or activation of alternative angiogenic pathways, thereby supporting resistance-informed treatment modification.

Collectively, the overlapping functions of humanization, multivalent and multispecific assembly, drug conjugation, molecular imaging, and Nb-CAR-T engineering provide broad coverage of key biological barriers, including affinity, specificity, tissue penetration, serum half-life, immune activation, and therapeutic resistance. Rather than defining a single universally optimal format, this modular integration expands the application of angiogenesis-targeting nanobodies across diagnostic imaging, sustained pathway inhibition, targeted drug delivery, immune modulation, and adoptive cell therapy.

## 5 Potential indications

Angiogenesis-targeting nanobodies, characterized by high tissue penetrability and engineering flexibility, have transitioned from an initial focus on oncology to ocular neovascular diseases. This shift highlights their potential for treating various other vascular-related disorders.

Early research primarily focused on the inhibition of tumor angiogenesis; for instance, the VEGF/Ang2 dual-targeting nanobody fusion protein, represented by BI 836880, has undergone phase I evaluation as monotherapy and in combination with PD-1 blockade in patients with advanced solid tumors. These studies established a recommended dose and demonstrated a manageable, although VEGF-pathway-related, safety profile, together with preliminary antitumor activity. Confirmatory trials are required to determine whether dual VEGF/Ang-2 blockade provides clinically meaningful advantages over existing anti-angiogenic strategies (94, 174). Concurrently, advancements in intraocular delivery systems and the favorable diffusion profiles of small-molecule antibody fragments have enabled breakthroughs for nanobodies in treating wet age-related macular degeneration (wAMD) and diabetic macular edema (DME). Their low molecular weight allows for efficient diffusion within the vitreous cavity. When combined with novel long-acting delivery technologies, nanobodies are expected to overcome the frequent dosing limitations of traditional anti-VEGF therapies. Notably, pathological neovascularization drives various chronic diseases, including rheumatoid arthritis, organ fibrosis, and ischemic injury, providing a biological rationale for expanding nanobody indications (175). In rheumatoid arthritis, VEGF/Ang-2-mediated synovial pannus formation is closely associated with bone erosion. Studies confirm that anti-VEGF antibodies effectively inhibit synovial pannus proliferation in arthritis models, suggesting translational potential for localized inflammation (176, 177). Similarly, inhibiting abnormal angiogenesis may alleviate fibrotic progression in liver and pulmonary fibrosis (10, 11). Furthermore, neovascularization critically influences the progression of conditions such as psoriasis, atherosclerosis, and vascular malformations (178, 179). It should be noted that most current studies on nanobody-based anti-angiogenic therapies have been conducted in oncology models. Extrapolation to non-oncological indications remains preliminary and will benefit from disease-specific pharmacokinetic and biodistribution studies. While anti-VEGF nanobodies have shown efficacy in suppressing synovial angiogenesis in preclinical arthritis models (180), the dense stromal composition of rheumatoid synovium and the absence of EPR effect may limit *in vivo* penetration compared to solid tumors. Similarly, the utility of nanobodies in fibrotic diseases remains hypothetical, as current data are restricted to mechanistic links between angiogenesis and fibrosis, with no direct evidence of nanobody accumulation in fibrotic neovasculature to date.

Similarly, it is important to note that current evidence on nanobody tissue penetration is heavily biased toward solid tumor models. The pathological neovasculature in non-neoplastic diseases exhibits distinct structural and functional characteristics. Exemplified by choroidal neovascularization (CNV) in wet age-related macular degeneration (AMD) and intraplaque neovessels in atherosclerosis, both vessels exhibit a strikingly immature phenotype and high permeability, however, CNV manifests as a subretinal membranous vascular plexus that breaches the outer blood–retinal barrier (181), whereas intraplaque neovessels comprise a disorganized meshwork of thin-walled, acellular (pericyte-deficient), non-muscular vessels confined strictly within the arterial wall (182). These differences can significantly affect nanobody bioavailability at non-tumor sites. Therefore, systematic comparative studies across relevant disease models are essential to accurately define the translational potential of nanobodies beyond oncology.

## 6 Clinical development status and translational considerations

The structural advantages and engineering versatility of nanobodies (Nbs) make them promising candidates for next-generation anti-angiogenic therapies. However, the developmental maturity of various Nb formats differ significantly, their successful clinical translation remains hindered by substantial challenges.

### 6.1 Current status of clinical evidence

To date, the clinical development of anti-angiogenic nanobodies (Nbs) has been driven primarily by multivalent and multispecific scaffolds, alongside molecular imaging probes (**Table 3**). In contrast, other engineered formats remain largely in the preclinical stage. Among nanobody constructs directly targeting angiogenic pathways, the most advanced candidate is BI 836880, a trivalent fusion protein that combines antagonistic VHHs against VEGF and Ang-2 with an albumin-binding domain to extend its half-life. Phase I/II trials (e.g., NCT03468426) in advanced solid tumors, including hepatocellular carcinoma (HCC) and non-small cell lung cancer (NSCLC), have demonstrated a manageable safety profile and preliminary anti-tumor activity (94, 95, 174). Furthermore, this therapeutic construct is actively being translated to ocular indications. Capitalizing on the small molecular weight of Nbs to enhance intravitreal diffusion, BI 836880 has entered clinical evaluation (e.g., NCT03861234) for ophthalmic diseases such as wet age-related macular degeneration (wAMD). While these ongoing studies represent a critical milestone for Nb applications in ophthalmology, long-term efficacy and safety comparisons against established intravitreal biologics remain to be fully elucidated.

**Table 3:** Current clinical development landscape of angiogenesis-related nanobody therapeutics and molecular imaging probes.

Candidate/modality	Target and clinical setting	Trial, phase, status, participants	Translational interpretation	References
BI 836880 monotherapy (intravenous)	VEGF-A/Ang-2/HSA; refractory advanced solid tumors	NCT02674152 and NCT02689505; Phase I; completed; n=29 and 24	Established dose, target engagement, and an early safety profile, but the nonrandomized dose-escalation cohorts cannot establish comparative efficacy, durability, or the clinical importance of repeated-dose immunogenicity.	(94, 95)
BI 836880 plus ezabenlimab (multinational)	VEGF-A/Ang-2 plus PD-1; multiple advanced or metastatic solid tumors	NCT03468426; Phase Ib; completed; n=252	Provides clinical support for vascular-checkpoint co-inhibition, but heterogeneous tumor cohorts, prior therapies, and the absence of randomized comparators preclude conclusions about superiority over current standards.	(183)
BI 836880 alone or plus ezabenlimab	VEGF-A/Ang-2 with or without PD-1; advanced solid tumors	NCT03972150; Phase I; completed; n=21	Supports dose feasibility and manageable safety in a Japanese population, but the sample was too small to define population-specific efficacy or uncommon toxicities.	(184)
BI 836880 (intravitreal)	VEGF-A/Ang-2; treatment-resistant wet age-related macular degeneration	NCT03861234; Phase I/IIa; completed; n=43	Demonstrates feasibility of local ocular administration. Efficacy, durability, optimal retreatment interval, and benefit relative to established anti-VEGF or VEGF/Ang-2 biologics remain unresolved.	(185)
^99m^Tc-cAbVCAM1-5 (SPECT tracer)	VCAM-1; inflammatory activity in atherosclerotic plaques	NCT04483167 (ATHENA); Phase I; registry status unknown; last known active, not recruiting; n=13	Clinical utility cannot yet be judged. Diagnostic accuracy, reproducibility, lesion-level histopathologic correlation, and renal radiation exposure require transparent reporting and validation.	(147)
[^68^Ga]Ga-NOTA-anti-CD206-sdAb (MMR Phase I)	CD206/MMR-positive macrophages; solid tumors by PET/CT	NCT04168528; Phase I/IIa; terminated; 13 registered, 7 reported	Provides strong pharmacokinetic proof of concept, but the cohort is insufficient for sensitivity, specificity, prognostic value, or treatment-selection claims. Renal, hepatic, and splenic uptake may limit some applications.	(148)
^68^GaNOTA-anti-MMR-VHH2 (MITRAS basket study)	CD206/MMR-positive macrophages; oncology, atherosclerosis, immune activation syndromes, and sarcoidosis	NCT04758650; Phase II; registry status unknown; last known recruiting; n=140 estimated	The broad basket design explores biological generalizability but introduces substantial indication and endpoint heterogeneity. Current registry staleness and incomplete public outcome reporting limit interpretation.	(149)
^68^GaNOTA-anti-MMR-VHH2 (MAGMA)	CD206/MMR-positive macrophages; resectable non-small cell lung cancer	NCT05933239; Phase II; recruiting; n=20 planned	The focused histopathology-correlative design directly addresses target-validation and quantification gaps, but clinical utility and outcome prediction will require larger, multicenter validation.	(186)

Nbs have achieved significant success in nuclear medicine. Several tracers have advanced to early-phase clinical trials, robustly validating their “rapid-clearance, high-contrast” pharmacokinetic paradigm. For instance, the VCAM-1-targeting ^99m^Tc-cAbVCAM1–5 has completed Phase I trials (NCT04483167) for evaluating atherosclerotic plaque inflammation (138). Early results indicate a favorable safety profile and specific lesion uptake; however, high renal retention remains a persistent challenge, potentially obscuring adjacent abdominal signals and increasing local radiation burden. Similarly, [^68^Ga]Ga-NOTA-anti-MMR-VHH2, which targets the macrophage mannose receptor, is undergoing Phase I/II trials (NCT04168528, NCT04758650) for atherosclerosis and oncology (140). Preliminary data confirm rapid target accumulation and excellent image contrast, yet managing the dosimetric limits associated with renal clearance continues to be a critical hurdle.

Conversely, Nb-CAR-T, NDCs and NDVs targeting angiogenesis remain confined to *in vitro* studies or animal models. The transition of these modalities from bench to bedside remains hindered by unique toxicological and pharmacological challenges. Specifically, the rapid systemic clearance of nanobodies curtails their therapeutic window, while structural modifications intended to extend half-life risk compromising their inherent tissue penetrability. Furthermore, for Nb-CAR-T therapies, overcoming T-cell exhaustion within the hypoxic tumor microenvironment and preventing “on-target, off-tumor” toxicities on healthy vasculature represent formidable barriers to clinical translation.

### 6.2 Critical translational challenges

The clinical translation of engineered nanobodies (Nbs) faces intrinsic biological limitations and extrinsic manufacturing hurdles that must be rigorously addressed to achieve widespread clinical adoption. A primary bottleneck involves pharmacokinetic trade-offs and renal handling. Albumin-binding domains, Fc fusion, PEGylation, PASylation, multimerization, and association with nanoparticles can reduce renal filtration and extend systemic exposure (119). However, these modifications increase hydrodynamic size and hinder deep tissue diffusion. Furthermore, high renal reabsorption leads to substantial kidney accumulation, yet the long-term tubular toxicity associated with chronic anti-angiogenic dosing remains insufficiently characterized.

From a resistance-oriented perspective, distinct mechanisms of vascular escape require mechanism-specific nanobody engineering. Incorporating domains against relatively stable vascular or stromal markers, including CD93, CD105, integrins, or fibronectin EDB, may further decrease reliance on dynamically regulated receptors such as VEGFR-2 (56). In contrast, vessel co-option and vasculogenic mimicry are unlikely to be adequately controlled by ligand neutralization alone (66). These mechanisms may require nanobody–drug conjugates or targeted delivery systems directed against tumor–vascular adhesion molecules, integrins, VE-cadherin, matrix metalloproteinases, or disease-associated extracellular matrix epitopes to inhibit perivascular invasion or eliminate tumor cells that form vascular-like channels (59). Metabolic and matrix-mediated resistance may be better addressed using hypoxia- or pH-responsive nanobody delivery platforms that release metabolic inhibitors, cytotoxic agents, or matrix-modulating payloads within resistant tumor regions (69). Immune-mediated resistance may be targeted using bispecific constructs that combine VEGF or Ang2 blockade with PD-L1 inhibition or the targeting of immunosuppressive myeloid populations (93, 174). Nanobody-based molecular probes may also enable longitudinal monitoring of vascular target expression, hypoxia, stromal remodeling, and immune-cell infiltration, thereby guiding the selection and timing of mechanism-matched therapies. Although these strategies are mechanistically plausible, most remain at the preclinical stage and require validation in orthotopic, metastatic, and treatment-relapse models.

Immunogenicity and long-term safety also present ongoing concerns. Although humanization mitigates immunogenic risk, it does not eliminate it entirely. The development of anti-drug antibodies (ADAs) can be triggered by key molecular features, including residual non-human framework residues, exposed neoepitopes, synthetic linkers, and albumin-binding modules (77). Furthermore, chemical conjugation sites, protein aggregation, product-related impurities, and repeated administration regimens all contribute to eliciting an unwanted immune response. Consequently, repeated-dose clinical programs should prospectively evaluate comprehensive immunogenicity profiles rather than relying solely on *in silico* T-cell epitope predictions. Such evaluations must systematically monitor baseline reactivity, treatment-emergent binding and neutralizing antibodies, altered pharmacokinetic exposure, hypersensitivity, loss of efficacy, and immune complex–mediated toxicities.

From a production standpoint, multispecific assemblies and site-specific nanobody-drug conjugates technologies often suffer from low yields and challenging scale-up processes. The lack of a universally established Chemistry, Manufacturing, and Controls (CMC) rules further complicates regulatory and formulation pathways. Thus, developers require tailored agency guidance to manage host cell protein clearance from microbial expression systems, ensure multi-domain stability, and optimize delivery formulations. Overall, engineered nanobodies represent a clinically credible but not uniformly mature platform. Successful translation will depend on precisely aligning molecular design, pharmacokinetics, manufacturability, and safety monitoring with the specific biological and therapeutic requirements of each indication.

## 7 Conclusion and future perspectives

Driven by rapid advancements in antibody engineering, precision delivery, and molecular imaging, nanobodies have emerged as a crucial platform for anti-angiogenic therapies due to their low molecular weight, high stability, excellent tissue penetrability, and modularity. Compared to traditional monoclonal antibodies, nanobodies more efficiently penetrate solid tumors, while their performance in other angiogenesis-dependent diseases (e.g., rheumatoid arthritis synovium, ocular neovascularization, or fibrotic tissues) requires further preclinical validation. Importantly, conventional monoclonal antibodies retain several clinically relevant advantages. FcRn-mediated recycling extends systemic circulation and sustains target exposure, thereby reducing dosing frequency and prolonging therapeutic activity. Their Fc domains can also engage antibody-dependent cellular cytotoxicity, antibody-dependent cellular phagocytosis, and complement-dependent cytotoxicity, which may be beneficial when immune effector recruitment or depletion of target-expressing cells is required. Accordingly, nanobodies should not be viewed as universal substitutes for conventional monoclonal antibodies. Instead, the two platforms offer complementary pharmacokinetic and functional profiles, nanobodies may be advantageous when rapid tissue penetration, access to spatially restricted epitopes, multispecific engineering, localized delivery, or molecular imaging is required.

Despite their considerable promise, the clinical development of angiogenesis-targeting nanobodies requires resolution of several fundamental scientific and technical challenges. Pathological angiogenesis is highly heterogeneous across diseases, patients, and disease stages and is dynamically shaped by endothelial plasticity, hypoxia, extracellular matrix remodeling, immune–vascular crosstalk, vessel co-option, and vasculogenic mimicry. It is therefore essential to distinguish disease-driving targets from transient or context-dependent vascular markers and to define the mechanisms. Integrating single-cell sequencing, spatial multi-omics, longitudinal molecular imaging, and analyses of patient-derived specimens may help identify vascular vulnerabilities. Major challenges encountered during the engineering design phase primarily include balancing extended serum half-life with tissue penetration, minimizing renal accumulation and immunogenicity, and optimizing the domain orientation, valency, linker design, stability, and pharmacokinetic properties of multivalent and multispecific constructs. For NDCs and NDVs, precise control of conjugation sites, drug-to-nanobody ratios, linker stability, payload release, carrier biodegradation, and long-term biodistribution will also be critical.

Additionally, manufacturing and regulatory considerations should be integrated from the earliest stages of nanobody development. Future manufacturing strategies should incorporate quality-by-design principles, high-throughput developability screening, process analytical technologies, site-specific conjugation, controlled molecular assembly, and scalable continuous or semi-continuous production. Regulatory frameworks must also address product heterogeneity, critical quality attributes, potency assays, immunogenicity, renal and long-term toxicity, process comparability, and interactions among the components of multifunctional products. Harmonized standards and early communication among researchers, manufacturers and regulatory authorities will be essential to reduce development uncertainty and ensure that molecular design, manufacturing strategy, and clinical evaluation progress in parallel. Furthermore, artificial intelligence and precision medicine are likely to shape the next phase of nanobody engineering and clinical application. Generative protein models, structure-prediction algorithms, and iterative active-learning systems may accelerate the development of humanized, multispecific, and *de novo* nanobodies against complex vascular targets; however, computational predictions must be validated through high-throughput experimental screening and clinically relevant disease models. In parallel, early-phase trials of angiogenesis-targeting nanobodies should incorporate pharmacodynamic biomarkers of target engagement and vascular normalization, longitudinal monitoring of blood pressure and proteinuria, renal function assessments, immunogenicity testing, and exposure–response analyses. Molecular imaging studies should move beyond demonstrating tracer uptake and include prespecified evaluations of diagnostic accuracy, reproducibility, effects on treatment selection, and impact on patient outcomes.

In conclusion, angiogenesis-targeting nanobodies are emerging as versatile platforms for vascular modulation, targeted delivery, molecular imaging, and immunotherapy. These advancements have yielded promising strategies primarily for the precise diagnosis and interventional treatment of solid tumors. Although emerging opportunities exist for some non-neoplastic vascular disorders, extensive preclinical validation is still required to establish their clinical utility and safety.
